# Prevalence of Gestational Diabetes Mellitus in the Middle East and North Africa, 2000–2019: A Systematic Review, Meta-Analysis, and Meta-Regression

**DOI:** 10.3389/fendo.2021.668447

**Published:** 2021-08-26

**Authors:** Rami H. Al-Rifai, Noor Motea Abdo, Marília Silva Paulo, Sumanta Saha, Luai A. Ahmed

**Affiliations:** ^1^Institute of Public Health, College of Medicine and Health Sciences, United Arab Emirates University, Al Ain, United Arab Emirates; ^2^Department of Community Medicine, R. G. Kar Medical College, Kolkata, India

**Keywords:** gestational diabetes mellitus, MENA region, prevalence, meta-analysis, systematic review

## Abstract

Women in the Middle East and North Africa (MENA) region are burdened with several risk factors related to gestational diabetes mellitus (GDM) including overweight and high parity. We systematically reviewed the literature and quantified the weighted prevalence of GDM in MENA at the regional, subregional, and national levels. Studies published from 2000 to 2019 reporting the prevalence of GDM in the MENA region were retrieved and were assessed for their eligibility. Overall and subgroup pooled prevalence of GDM was quantified by random-effects meta-analysis. Sources of heterogeneity were investigated by meta-regression. The risk of bias (RoB) was assessed by the National Heart, Lung, and Blood Institute’s tool. One hundred and two research articles with 279,202 tested pregnant women for GDM from 16 MENA countries were included. Most of the research reports sourced from Iran (36.3%) and Saudi Arabia (21.6%), with an overall low RoB. In the 16 countries, the pooled prevalence of GDM was 13.0% (95% confidence interval [CI], 11.5–14.6%, *I^2^*, 99.3%). Nationally, GDM was highest in Qatar (20.7%, 95% CI, 15.2–26.7% *I^2^*, 99.0%), whereas subregionally, GDM was highest in Gulf Cooperation Council (GCC) countries (14.7%, 95% CI, 13.0–16.5%, *I^2^*, 99.0%). The prevalence of GDM was high in pregnant women aged ≥30 years (21.9%, 95% CI, 18.5–25.5%, *I^2^*, 97.1%), in their third trimester (20.0%, 95% CI, 13.1–27.9%, *I^2^*, 98.8%), and who were obese (17.2%, 95% CI, 12.8–22.0%, *I^2^*, 93.8%). The prevalence of GDM was 10.6% (95% CI, 8.1–13.4%, *I^2^*, 98.9%) in studies conducted before 2009, whereas it was 14.0% (95% CI, 12.1–16.0%, *I^2^*, 99.3%) in studies conducted in or after 2010. Pregnant women in the MENA region are burdened with a substantial prevalence of GDM, particularly in GCC and North African countries. Findings have implications for maternal health in the MENA region and call for advocacy to unify GDM diagnostic criteria.

**Systematic Review Registration:**

PROSPERO CRD42018100629

## Introduction

Gestational diabetes mellitus (GDM) (1) is usually diagnosed during the second and third trimesters of pregnancy (2). Risk factors of GDM include excessive body weight, low level of physical activity, consanguineous marriage, previous history of GDM, glycated hemoglobin >5.7%, and history of cardiovascular disease (3). As the toll of overweight and obese reproductive-age females soars, the risk of developing hyperglycemia in pregnancy increases (4).

GDM has a global public health burden (5) with both short- and long-term consequences on health. The short-term ramifications of GDM include adverse perinatal outcomes for the affected women (e.g., preeclampsia, polyhydramnios, and increased cesarean section [“C-section”] risk) and their neonates (e.g., macrosomia and shoulder dystocia) (1, 6), whereas the long-term complications of GDM incorporate the risk of type 2 diabetes mellitus (T2DM) for the mother and the risk of childhood obesity, impaired glucose tolerance, and/or metabolic syndrome for their neonates (6). Since increased blood glucose levels are associated with certain perinatal complications, gestational blood glucose control is vital (7).

Understanding population-specific healthcare needs at specific points of time is essential, and prevalence estimates are ideal for such purposes (8). Unfortunately, the global GDM prevalence estimates (<1%–28%) show a wide variation due to ethnicity, ethnic variation among various populations, and inconsistent use of screening and diagnostic criteria (4, 9). To precisely estimate the burden of GDM of a particular geographic area, it is essential to determine the region-specific prevalence estimate. There is scant literature on the prevalence of GDM in the Middle East and North Africa (MENA) region, although two of the main risk factors [physical inactivity and above-normal body mass index (BMI)] are identified as being highly prevalent in this region (10). Moreover, three of the world’s top ten most prevalent countries for diabetes mellitus belong to this region: Saudi Arabia (24%), Kuwait (23%), and Qatar (23%) (11). For the entire Eastern Mediterranean region, the existing prevalence estimate of GDM is 14.5%, although this includes only cases diagnosed according to the World Health Organization (WHO) 1999 criteria (4). One previous survey showed that physicians and hospitals in this region use different criteria to diagnose GDM (12).

A systematic review and meta-analysis of prevalence studies is considered to be an ideal method to understand the burden of GDM at regional and national levels. In this systematic review, meta-analysis, and meta-regression, we estimated the weighted pooled prevalence of GDM in the MENA region, at the regional, subregional, and national levels, based on literature published between January 2000 and December 2019.

## Methods

This review follows the Preferred Reporting Items for Systematic Review and Meta-Analysis (PRISMA) 2009 guidelines (13). The PRISMA checklist is provided elsewhere (**Supplementary File 1**). Following our published protocol, we report here “systematic review 2” (14). We implemented minor amendments whenever needed, including an updated database search.

### Data Source and Searches

To identify eligible studies reporting the prevalence of GDM in the MENA countries, we conducted a comprehensive search of five electronic databases (MEDLINE, EMBASE, Web of Science, SCOPUS, and Cochrane library) from January 1, 2000, to December 31, 2019, using variant Medical Subject Headings and free-text terms. Restricting the literature search to 2000 was to estimate changes in the GDM prevalence over the past two decades (before and after 2010), at national, sub-regional, and regional levels, whenever enough data is available for the meta-analysis. The literature search strategy was developed in consultation with an expert librarian at the National Medical Library at the United Arab Emirates University. The full search strategy available in the published protocol (14). Retrieved references were imported to the Covidence software (Covidence, Melbourne, Australia) (15). Deduplication of similar references was performed automatically by the Covidence software.

### Study Selection

To identify and select studies for inclusion, we followed the PECO(T) framework: participants, exposure, comparator, outcome(s), and type of study (16). However, we considered only participants and outcomes because the focus of this review was on studies reporting the prevalence of GDM. Study eligibility criteria are presented in **Table 1**.

**Table 1:** Study eligibility criteria.

Criteria	Inclusion	Exclusion
Population	Pregnant women regardless of their age, parity, or any maternal or sociodemographic characteristics	Non-pregnant women
Outcome	Studies reported quantitative or calculable GDM prevalence estimate(s) regardless of the GDM diagnostic criteria/guidelines or pregnancy trimester	Studies on pregnant women with no information related to GDM prevalence
Sample size	Studies with at least ten pregnant women tested for GDM	Studies with less than ten pregnant women tested for GDM
Study design	Cross-sectional, cohort studies, case–control studies comparing no-GDM with no-GDM subpopulations, and trials with nonpharmaceutical interventions	Case–control studies comparing GDM with no-GDM populations, qualitative studies, modeling studies, case reports and case series regardless of the number of cases, narrative and systematic reviews, conference abstracts with no full information, editorials, commentaries, letters to the editor, author replies, and other publications that did not include quantitative data on the prevalence of GDM
Geographical region	Any of the 18 Arab countries (Algeria, Bahrain, Djibouti, Egypt, Iraq, Jordan, Kuwait, Lebanon, Libya, Morocco, Oman, Qatar, Saudi Arabia, Syria, Tunisia, United Arab Emirates, West Bank and Gaza, and Yemen) in addition to Iran and Malta in the MENA region, according to the definition of the World Bank Country and Lending Groups (17).	All other countries
Publication period	January 2000 to December 2019	Studies conducted before January 2000 or after December 2019 and studies for which the time period of the GDM tests in pregnant women was unclear
Language	English language	Non-English studies
Setting	No limitations. Hospital based, population based, or clinic based.	No limitations
Duplicate studies	–	Studies duplicating or potentially duplicating GDM ascertainment in the same population. In the case of duplicate publications, we included only the study containing the most relevant information in the context of the prevalence of GDM

### Identifying Eligible Studies

**Figure 1:**
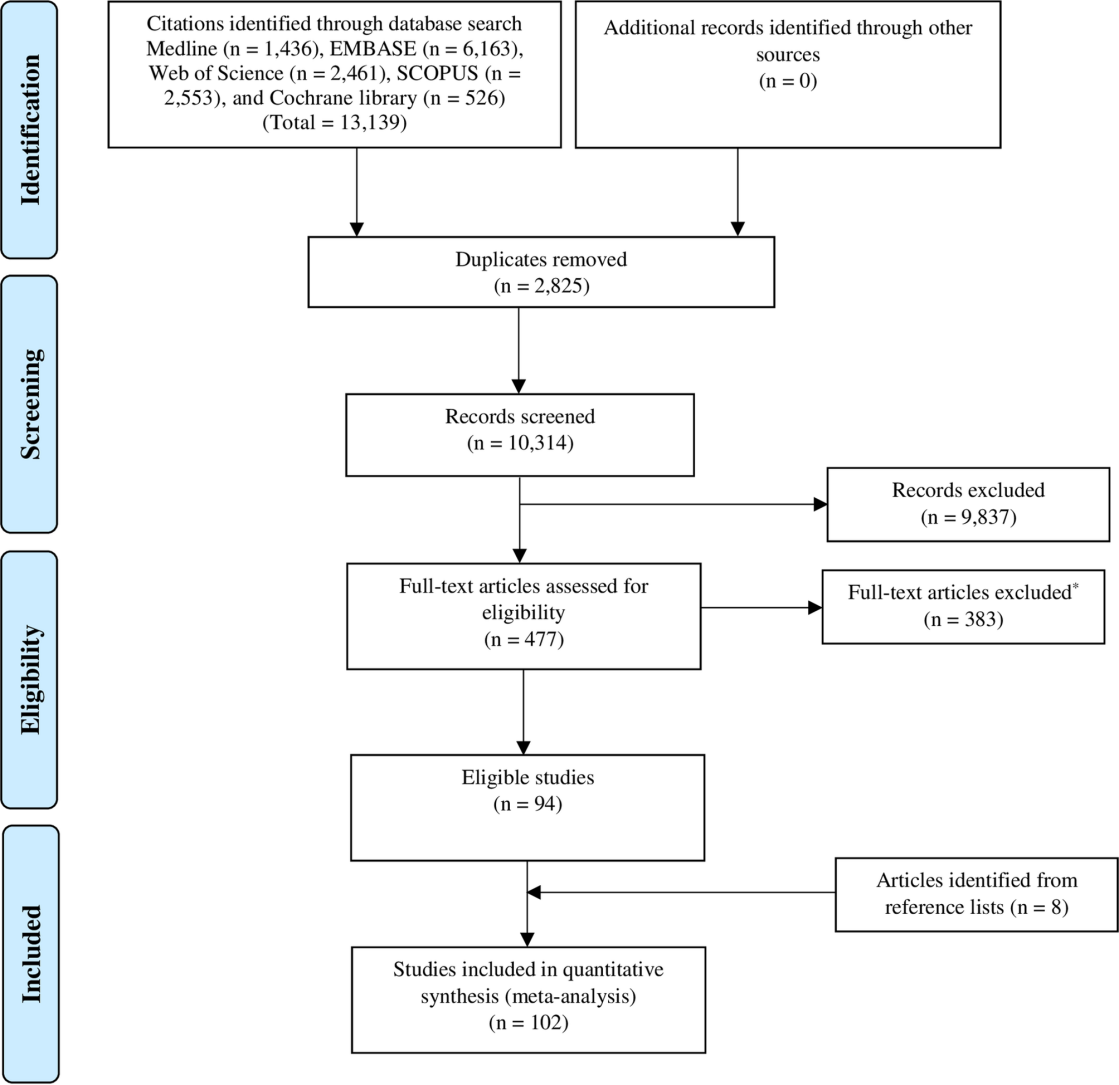
PRISMA flowchart of study selection.

Titles and abstracts were screened by RHA, NMA, and MSP to detect eligible research reports on the prevalence of GDM. For studies that appeared eligible, the full text was reviewed (RHA, NMA, and MSP). Screening of all titles and abstracts and full text articles was performed independently by two reviewers. Disagreements among reviewers were resolved by discourse. We also searched the reference lists of eligible studies for studies that might have been missed. **Figure 1** shows the PRISMA flowchart of study selection.

In this review, the term “research report” is used to refer to a full published research document. The term “study” is used to refer to a single study on a specific population group. One big observational study (one research report) provides GDM data stratified into four age groups (four studies). Hence, one research report could contribute several studies on GDM prevalence.

### Data Extraction and Quality Assessment

Relevant data from eligible studies were extracted into a predesigned Excel sheet using a predefined list of numerical and string variables. The outcome of interest was the weighted prevalence of GDM in pregnant women in the MENA countries, according to various characteristics including, but not limited to, age, BMI, trimester, and time period. We extracted author names, publication year, country, city, and study setting. In addition, data on the implemented methodology (design, data collection period, sampling strategy, and GDM diagnosis and ascertainment methodology) and characteristics of the studied pregnant women (age, pregnancy trimester, sample size, number of women with GDM and GDM prevalence) were extracted whenever available.

In addition to the overall prevalence of GDM, some research reports also reported the prevalence of GDM stratified according to different characteristics, such as age, parity, comorbidity, pregnancy trimester, and BMI. In such reports, data extraction was performed for the stratified GDM prevalence, following the rule that the study had to have at least ten tested subjects per strata; otherwise, information on the entire tested sample was extracted. A predefined sequential order was established when extracting stratified GDM prevalence estimates as follows: GDM stratified first according to comorbidities followed by parity, age, and BMI. This prioritization was used to identify the strata with more information on the tested pregnant women. When there was no stratification for the prevalence of GDM, we extracted the overall GDM prevalence measured.

For each research report reporting the stratified prevalence of GDM according to more than one category (i.e., age and BMI), one category per research report was considered and included based on the aforementioned prioritization scheme, to avoid double counting. In studies in which GDM was ascertained using different guidelines, the most sensitive and reliable ascertainment assay was considered (i.e., prioritizing fasting blood glucose over self-reported) or was based on the most recent and updated criteria (i.e., prioritizing WHO 2010 over 2006 criteria).

The risk of bias (RoB) assessment was performed at the level of the research report rather than the study. The quality of each research report was evaluated according to criteria of the National Heart, Lung, and Blood Institute (18). Six of 14 items from the quality assessment tool for prevalence studies were used (18). The six quality-related items assessed the research question/objectives, studied population, sample size justification, and outcome measures and assessment. Eight items were not used because they are applicable only to follow-up cohort studies. For additional quality assessment, we also assessed the robustness of the implemented methodology using three additional quality-of-evidence criteria: sampling methodology, GDM ascertainment methodology, and precision of the estimate. Studies were considered to have “high” precision if at least 100 women were tested for GDM. We computed the overall proportion of research reports with potentially low RoB across each of these nine quality criteria and also computed the proportion (out of nine) of quality items with a potentially low RoB for each of the included research reports.

Data abstraction and quality assessment were performed independently by two reviewers (NA and MP) and cross-checked for disagreements. Any discrepancies in the extraction phase or in the quality assessment between the reviewers were discussed and resolved with a consultation of a senior reviewer (RA-R).

### Data Synthesis and Analysis

To estimate the weighted pooled prevalence of GDM and the corresponding 95% confidence interval (CI), we performed meta-analyses of the extracted data. The Freeman–Tukey double arcsine transformation method was applied to stabilize the variances of the prevalence measures (19). The inverse variance method was used to weight the estimated pooled prevalence measures (20). Dersimonian–Laird random-effects model was used to estimate the overall pooled GDM prevalence (21). Cochran’s Q statistic and the inconsistency index, *I* ^2^, were calculated to measure heterogeneity (22). Along with the pooled estimates, ranges and median were also reported to describe the dispersion of the GDM prevalence measures reported in the literature. The prediction interval, which estimates the 95% interval in which the true effect size in a new prevalence study will lie, was also quantified and reported (22).

For the subgroup meta-analysis, country-level pooled estimates were generated overall and based on time period. In addition, to estimate the change in GDM both at the country level and overall, the data collection period was stratified into two time periods: 2000–2009 and 2010–2019. For studies in which the data collection period overlapped, the collection period was defined as “overlap” so as not to miss any important data when estimating country-level, subregional, and regional prevalence. The median (~2 years) was used in studies with an unclear data collection period. In these studies, the median was subtracted from the year of publication to estimate the year of data collection.

The weighted pooled prevalence, regardless of country, was also estimated according to the age of the pregnant women, trimester, BMI, study period, GDM ascertainment guidelines, and sample size (<100 or ≥100). The provision of pooled estimates regardless of the ascertainment guidelines was justified by the fact that the women were defined and treated as GDM patients following each specific ascertainment guideline.

Accumulated evidence has shown that GDM is associated with an increased risk of C-section (23, 24) and maternal mortality (4). Independent of the research report and the characteristics of the tested pregnant women for GDM, we estimated the pooled GDM prevalence according to the C-section rate and maternal mortality ratio (MMR). Information on the C-section rate (25, 26) and MMR were retrieved from various resources (27). Depending on data availability, information on C-section rate and MMR was extracted in the same or the closest year to the estimated GDM prevalence. For every GDM study, the rate of C-section was then categorized as <15%, 15–29%, >30%, or unclear, whereas the MMR was categorized as either ≤100/100,000 live births, >100/100,000 live births, or unclear.

To provide prevalence estimates at a subregional level, we regrouped MENA countries into four subregions, namely, North Africa, Gulf Cooperation Council (GCC) countries, Levant, and Iran/Iraq region. We estimated the overall pooled prevalence in these subregions and according to patient age, trimester, BMI, study period, GDM ascertainment guidelines, rate of C-section, and MMR.

Random-effects univariate and multivariable meta-regression models were implemented to identify sources of between-study heterogeneity and to quantify their contribution to variability in the prevalence of GDM. In univariate meta-regression models, analysis was performed by country, age, pregnancy trimester, BMI, and sample size. All variables with a *p-*value <0.1 in the univariate models were included in the multivariable model. In the final multivariable model, a *p-*value ≤0.05 was considered statistically significant, which contributed to the heterogeneity in prevalence estimates.

### Publication Bias

A funnel plot was generated to explore the small-study effect on the pooled GDM prevalence estimates. The funnel plot was created by plotting each GDM prevalence measure against its standard error. The asymmetry of the funnel plot was tested using Egger’s test (28).

All analyses were performed using the *metaprop* (29) and *metareg* packages in Stata/SE v15 (30).

The study is registered with PROSPERO, number CRD42018100629.

## Results

### Database Search and Scope of the Review

Of the 13,139 citations retrieved from the 5 databases, 102 research reports were deemed eligible and included in this review (**Figure 1**).

The research reports were from 16 countries in the MENA region: Algeria (one), Bahrain (two), Egypt (four), Iraq (three), Iran (37), Jordan (four), Lebanon (two), Libya (one), Morocco (one), Oman (five), Qatar (six), Saudi Arabia (22), Sudan (two), Tunisia (one), United Arab Emirates (UAE) (eight), and Yemen (one). The prevalence data for both decades (time periods) were available from six countries (Bahrain, Iran, Oman, Qatar, Saudi Arabia, and the UAE); for the other countries, data were available for the time period 2010–2019 (**Table 2**). Self-reported GDM status was documented in five research reports (31, 73, 83, 90, 119). The predominantly used GDM diagnostic criteria in the MENA region were from the American Diabetes Association and the International Association of Diabetes and Pregnancy Study Group (ADA/IADPSG; 48.5% of studies).

**Table 2:** Summary of the included studies reporting the prevalence of GDM in pregnant women in the MENA region, 2000–2019, stratified by country (102 reports with 198 prevalence measures).

Author, year [Ref]	Duration of data collection	Country, city	Setting	Design	Sampling	Population	Strata	Ascertainment method	Tested sample	GDM	
										Positive	%
Tebbani F. et al. (31)	12/2013–12/2015	Algeria, Constantine	Maternities, antenatal and private gynecologists	PC	Unclear	Algerian pregnant women aged 19–41 years who entered prenatal care before 16 weeks of amenorrhea	All	Face-to-face interview	200	6	3.0
Rajab K. et al. (32)	2002–2010	Bahrain	Government central hospital that is responsible for approximately 80% of all births in Bahrain	CS	Whole population	Pregnant women	All 2002–2010	NDDG 1979 guidelines	49,552	4,982	10.1
Al Mahroos S. et al. (33)	1/2001–12/2002	Bahrain	ANC clinics at health centers and at Salmaniya Medical Complex	CS	All women during the study period	Nondiabetic pregnant women	All	Fourth International Workshop-Conference on GDM	10,495	1,394	13.7
							Bahraini		7,575	1,175	15.5
							Expatriate		2,920	219	7.5
Rakha S and El Marsafawy H (34)	01/2011 – 01/2019	Egypt, Mansoura	Pediatric cardiology unit in Mansoura University Children’s Hospital	CS	Whole population	Pregnant with at least one high risk indication of fetal echocardiography	All	Unclear	458	57	12.5
Rezk M and Omar Z (35)	05/2012–05/2017	Egypt	Shibin El-Kom	PS	Whole Population	Pregnant women with chronic HCV infection	All	Unclear	342	90	26.3
						Pregnant women with no HCV infection			170	10	5.9
Maged AM. et al. (36)	01/2011–02/2013	Egypt, Cairo	Kasr El Aini Hospital	PS	Unclear	Pregnant women in their first trimester with a singleton living fetus, excluding women with preexisting type 1 or 2 diabetes mellitus, hypertension, liver disease, renal disease, or the presence of active infection	All	ADA 2002	269	27	10.0
Elkholi DGEY and Nagy HM (37)	3/2007–3/2013	Egypt, Tanta	Infertility Clinic, Tanta University Hospitals	CS	Unclear	Obese pregnant women (BMI ≥30 kg/m^2^) with PCOS before treatment for infertility, attending 100 patients with android obesity and 100 patients with gynoid obesity	All	Fifth International Workshop Conference on Gestational Diabetes criteria	131	10	7.6
			Outpatient Clinic of Department of Obstetric			Non-PCOS pregnant women with android obesity were controls for group 1 and 100 non-PCOS pregnant women with gynoid obesity who were free of DM before pregnancy			177	14	7.9
Mohammed AK and Alqani VHA (38)	06/2016–07/2017	Iraq, Al-Diwaniyah	Child and Maternity Teaching Hospital	CS	Unclear	Pregnant women with a mean age of 30.02 ± 6.37 years	All	Unclear	49	12	24.5
Alawad ZM and Al-Omary HL (39)	09/2018–12/2018	Iraq, Baghdad	Baghdad teaching hospital	PC	Unclear	Women between 18 and 40 years of age, normal vaginal deliveries to live singletons with no congenital anomalies, women with normal thyroid function test	All	Unclear	35	7	20.0
Safari K et al. (40)	10/2017–01/2018	Iraq, Erbil	Hawler Maternity Teaching Hospital	CC	Unclear	Singleton Muslim pregnant women aged 18–35 years who fasted in Ramadan during the second trimester	All	Unclear	155	4	2.6
									144	12	8.2
Maghbooli Z et al. (41)	2005	Iran, Tehran	Five university hospital clinics of the Tehran University of Medical Sciences	CS	Unclear	Pregnant women with no previous history of DM and who sought prenatal care during the first half of their pregnancies	All	Carpenter and Coustan criteria	741	52	7.0
Abolfazl M et al. (42)	2006	Iran, Shiraz	Shiraz Hospital	Unclear	Random	Pregnant women with a mean age of 31.2 years	All	Unclear	420	70	16.6
Keshavarz M et al. (43)	12/1999–01/2001	Iran, Shahrood	Fatemiyeh Hospital	PC	Consecutive	All pregnant women within the catchment area of the hospital were referred to this antenatal service; twin pregnancies, miscarriages, terminations, and women with preexisting diabetes were excluded from our study	All	Carpenter and Coustan criteria	1,310	63	4.8
Hadaegh F et al. (44)	3/2002–3/2004	Iran, Bandar Abbas	Obstetrics clinics in various parts of Bandar Abbas city in southern Iran	CS	All women during the study period	Pregnant women with a mean age of 24.9 years in the 24th to the 28th week of pregnancy excluding women with history of diabetes, using drugs that affect glucose metabolism, with chronic liver disease, endocrine disorders (such as hyperthyroidism), or connective tissue disorders, and with major medical conditions, such as persistent hypertension	All	Carpenter and Coustan criteria	700	62	8.9
							<20 years		93	2	2.2
							20–24 years		279	15	5.4
							25–29 years		184	22	12.0
							30–34 years		103	13	12.6
							35–≥45 years		41	10	24.3
Amooee S et al. (45)	2006–2008	Iran, Sheraz	Hafez and Zeinabieh Hospitals of Shiraz University of Medical Sciences	CS	Unclear	All singleton pregnancies with and without minor β-thalassemia	With minor β-thalassemia	Unclear	510	16	3.5
							Without minor β-thalassemia		512	20	20.0
Lamyian M et al. (46)	08/2010– 01/2011	Iran, Tehran	Prenatal clinics in five hospitals affiliated with universities of medical sciences in different districts	PS	Random	Singleton pregnant women age 18–45 years, excluding preexisting diabetes and smokers	All	ADA 2016	1,026	71	6.9
Soheilykhah S et al. (47)	2007–2009	Iran, Yazd	Two prenatal clinics in Yazd	PS	Unclear	Iranian pregnant women with a mean age of 27 years, excluding those with prepregnancy DM	All	ADA 2004	734	95	13.0
							<25 years		247	19	7.7
							25–29 years		202	30	14.9
							≥30 years		285	46	16.1
Pirjani R et al. (48)	2012–2013	Iran, Tehran	Dr Shariati and Arash Hospitals	PS	Convenience	Pregnant women with a mean age of 28.70 ± 5.57 years (range 17–44 years) excluding women with a history of diabetes (type 1 or 2), tested for GDM at the 24th–28th weeks of pregnancy	All	ADA 2012	256	78	30.5
Soheilykhah S et al. (49)	01/2010–02/2013	Iran, Yazd	Two prenatal clinics (Mojibian and Shahid Sadoughi Hospitals	CS	Unclear	Pregnant women tested for GDM at 24–28 weeks of pregnancy, excluding women with type 1 or 2 diabetes, malignancies, acute or chronic inflammatory or infective diseases, acute or chronic liver disease, and iron deficiency anemia	All	ADA 2013	1,279	281	21.9
Shahbazian H et al. (50)	08/2014–02/2015	Iran, Ahvaz	Prenatal clinic of a public medical hospital and four private prenatal clinics	PS	Unclear	Pregnant women tested for GDM between 24 and 32 weeks of gestation	All	IADPSG	750	224	29.9
							15–24 years		190	32	16.8
							25–34 years		452	145	32.1
							35–44 years		108	47	43.5
Yassaee F et al. (51)	10/2008–2/2010	Iran, Tehran	Teaching hospital in the North of Tehran	PS	Unclear	Pregnant women with idiopathic thrombocytopenic purpura at a mean age of 28.9 years		Unclear	21	6	28.6
Ashrafi M et al. (52)	2012–2013	Iran, Tehran	Reproductive biomedicine research center, Royan Institute	CS	Unclear	Non-PCOS pregnant women who conceived spontaneously with a mean age of 26.4 years	All	Fifth International Workshop on GDM	234	17	7.3
						Non-PCOS pregnant women conceived with RT with a mean age of 30.7 years	All		234	70	29.9
						PCOS pregnant women with ART with a mean age of 29.6 years	All		234	104	44.4
Goshtasebi A et al. (53)	8/2010–1/2011	Iran, Tehran	Prenatal clinics in five hospitals affiliated with universities of medical sciences	CS	Consecutive	Pregnant women aged 18–45 years, singleton pregnancy, gestational age ≤6 weeks, gestations ≤2, and nonsmokers	All	ADA 2016	1,026	71	6.9
Ashrafi M et al. (54)	11/2011–10/2012	Iran, Tehran	Reproductive Biomedicine Research Centre of the Royan Institute,	CS	Unclear	Pregnant women who conceived after fresh IVF/ICSI or intrauterine insemination at a mean age of 31.3 years with no history of DM, family history of DM, GDM	All	ADA 2005	145	54	15.7
			Akbarabadi Women’s Hospital, affiliated with Tehran University of Medical Science	CS	Unclear	Pregnant women with singleton spontaneous pregnancies at a mean age of 26.6 years and with no history of DM, family history of DM, or GDM	All		215	22	25.1
Jamali S et al. (55)	4/2012–10/2015	Iran, Jahrom	Paymaneh Hospital Jahrom, Iran	CS	Unclear	Inclusion criterion was all women aged 15–45 years; incomplete and doubtful data were excluded; the study compared 154 women in the first group (teenage group), 400 women in the second group (control group), and 196 women in the third group (adult women)	All 15–45 years	Medical Records
									750	16.2	2.1
							15–19 years		154	1	0.6
							20–34 years		400	7	1.8
							35–45 years		196	8	4.1
Pourali L et al. (56)	7/2009–7/2014	Iran, Mashad	Ghaem Hospital	CS	Convenience	Women with dichorionic spontaneous twin pregnancy with a mean age of 27.1 years	All	Medical records	96	8	8.3
						Women with dichorionic pregnancy following ART with a mean age of 28.9 years			31	8	25.8
Mehrabian F and Rezae M (57)	1/2009–3/2013	Iran, Isfahan	Shahid Beheshti Hospital	CS	Unclear	Pregnant women who were infertile due to PCOS with an age range of 18–42 years	All	ADA 2011	180	50	27.8
Mehrabian F and Hosseini SM (58)	2011–2012	Iran, Isfahan	Isfahan University of Medical Sciences	CS	Convenience	Pregnant women without preexisting diabetes, mean age 27.6 years	All	Unclear	944	72	7.6
Hosseini E et al. (59)	10/2015–01/2017	Iran, Isfahan	10 community health care centers	CS	Consecutive	Women 18–45 years old with singleton pregnancy	All	IADSPG two-step approach	929	93	10.0
Hantoushzadeh S et al. (60)	2/2012–3/2015	Iran, Tehran	Maternal, Fetal and Neonatal Research Center, Vali-asr Teaching Hospital	CS	Unclear	Pregnant women aged 20–32 years with singleton pregnancies screened for GDM at 28 weeks. excluding women with a history of type 1 or type 2 diabetes mellitus, missing information about prepregnancy diabetes status or BMI, incomplete data on glucose tolerance testing or weight gain during pregnancy	All	ACOG	1,279	100	7.8
							Underweight		27	0	0.0
							Normal weight		751	45	3.3
							Overweight		381	35	9.2
							Obese		120	20	16.7
Niromanesh S et al. (61)	2008–2010	Iran, Tehran	Tehran Women General Hospital	CS	Consecutive	Normal pregnant women 20–35 years of age with gestational age 16–20 weeks, gravid >2, BMI of 20–25 kg/m² were included in the study, excluding women with a history of PTB, preeclampsia, diabetes, GDM, primigravida, those with a BMI >25, and high maternal age (>35 years)	High triglyceride level (>195 mg/dL)	Unclear	45	9	20.0
							Normal triglyceride level (<195 mg/dL)		135	8	5.9
Vaezi A et al. (62)	2009–2012	Iran, Tehran	Akbarabadi Hospital	RC	Convenient	Medical records of pregnant women aged between 18 and 50 years admitted to the hospital to obtain prenatal care	All	Unclear	580	56	9.6
							With asthma		274	37	13.5
							Without asthma		306	19	6.2
Hossein–Nezhad A et al. (63)	Unclear	Iran, Tehran	Five teaching hospitals affiliated with Tehran University of Medical Sciences	CS	Consecutive	Pregnant women referred to ANC visits with no known history with known diabetes were excluded from the study	All 15–45 years	Carpenter and Coustan
									2,416	114	4.7
							15–24 years		1,209	27	2.2
							25–34 years		1,001	56	5.6
							35–45 years		206	31	15.0
Nastaran SA et al. (64)	10/2009–8/2010	Iran, Tehran	Milad Hospital	PS	Convenience	Pregnant woman referred to the pregnancy care clinics with a single fetus, aged 18–35 years with a gestational age of 1–13 weeks, a parity of 3 or less, lack of known systemic diseases, and lack of gestational diabetes during previous pregnancies	All	Carpenter and Coustan	600	49	8.2
Talebian A et al. (65)	2/2007–12/2012	Iran, Kashan	Shabihkhani, Shahid Beheshti and Milad hospitals	CS	Unclear	Pregnant women with normal pregnancies and with neural tube defects	All	Unclear	300	21	7.3
Kouhkan A, et al. 2018 (66)	11/2014–1/2017	Iran, Tehran	Royan Institute and maternity teaching hospital located in Tehran	PC	Whole population	Singleton pregnant women aged 20–42 years, who conceived via ART or SC	All	ADA/IAPDSG	574	287	50
Abedi P et al. (67)	08/2013–10/2014	Iran, Ahfav	Four centers from the east and three centers from the west of Ahvaz	CS	Unclear	Pregnant women	All	Medical records	700	43	6.1
Pezeshki B et al. (68)	04/2015–04/2016	Iran, Zanjan	Seven health care centers affiliated with Zanjan University of Medical Sciences	PC	Whole population	Pregnant women between the ages of 18 and 35 years, gestational age of equal or less than 12 weeks at first visit, a BMI of between 30 and 18.5 kg/m^2^, and a blood pressure of less than 140/90 mm Hg during first visit, tested for GDM in the first trimester	All	ADA 2016	356	25	7.0
Heydarpour F et al. (69)	2015–2017	Iran, four cities were selected from each province	One rural and one urban health clinic were selected in each city	RC	Multistage	Pregnant women with: a hemoglobin level less than 11 g/dL during the first trimester	All	Medical records	1,038	27	2.6
						a hemoglobin level more than 11 g/dL during the first trimester			2,463	106	4.3
						a hemoglobin level less than 11 g/dL during the third trimester			756	28	3.8
						a hemoglobin level more than 11 g/dL during the third trimester			1,986	68	3.4
Fazel N et al. (70)	08/2014–04/2015	Iran, Sabzevar	From 18 obstetric clinics associated with Mobini Hospital	PC	Cluster random sampling	Pregnant women in gestational week 24 or less	All	Medical records	1603	30	1.87
Nouhjah S. et al. (71)	03/2015–01/2016	Iran, Ahvaz	25 urban and public and private prenatal care clinics	PC	Unclear	Pregnant women	All	IADPSG	800	176	22.0
Maghbooli Z et al. (72)	04/2016–03/2017	Iran, Tehran	Prenatal care clinics in two regions in Tehran, Iran	CC	Unclear	Pregnant women living in nonpolluted areas	All	Unclear	44	3	6.8
Salehi-Pourmehr H et al. (73)	12/2012–01/2016	Iran, Tabriz	All health centers in Tabriz (65 centers and subcenters)	PC	Unclear	Obese (BMI ≥ 35 kg/m^2^) pregnant women in the first trimester of pregnancy, aged 18–35 years	All	Self-reported	62	7	11.0
Zargar M et al. (74)	2011–2016	Iran, Ahvaz	Pregnant women referring to three infertility centers in Ahvaz city	CC	Randomly	All women undergoing ART	All	Unclear	318	33	10.4
Mojtahedi SY et al. (75)	04/2010–05/2016	Iran, Tehran	Ziaeean and Imam Khomeini hospitals in Tehran	CS	Random	Mothers of neonates (<15 days) with hyperbilirubinemia (> 15 mg/dL)	All	Medical records	163	41	25.2
Eslami E et al. (76)	07/2016–04/2016/ 12/2017–02/2017	Iran, Tehran	12 health centers of Tehran	RCTs	Unclear	Singleton pregnant females with BMI greater than 25 aged 18 and older, gestational age of 16–20 weeks	All	Unclear	70	17	24.3
						Singleton pregnant females with BMI greater than 25, aged 18 and older, gestational age of 16–20 weeks receiving lifestyle training	All		70	15	21.4
Mardani M et al. (77)	2015–2016	Iran	Health care centers	CC	Whole population	Pregnant women with severe acute respiratory illness	All	Medical records	24	3	12.5
					Randomly	Living pregnant women with severe acute respiratory illness	All		100	4	4.0
Basha S et al. (78)	01/2015–01/2016	Jordan	Jordan University Hospital	CS	Consecutive	Women with singleton pregnancies tested for GDM at 24–28 weeks of pregnancy	All 15–49 years	IADPSG	644	87	13.5
							15–29 years		301	24	8.0
							30–39 years		302	50	16.5
							40–49 years		41	13	31.7
Abdel Razeq NM et al. (79)	2012/2013	Jordan	Nationwide in 18 maternity hospitals	CS	Unclear	All women who gave birth to dead or live neonates at 20 or more weeks of gestation	All	Medical records	21,075	253	1.2
Clouse K et al. (80)	04/2015–05/2015	Jordan, Amman	Al-Bashir Hospital	CS	Unclear	Pregnant women	All	Medical records and interviews	200	3	1.5
Khader YS et al. (81)	03/2011–04/2012	Jordan, nationwide	18 hospitals with maternity departments in three regions of Jordan (South, Middle, and North)	CS	Whole population	Deliveries with a gestational age ≥20 weeks	All	Medical records and interviews	21,928	261	1.2
Zein S et al. (82)	12/2012–11/2013	Lebanon, Beirut	Bahman hospital	CS	Unclear	Singleton pregnancies, nonanemic, having first prenatal visit before 12 weeks	All	IADPSG	104	16	15.4
Ghaddar N et al. (83)	09/2016–08/2017	Lebanon, Beirut and South Lebanon	Outpatient clinic of obstetrics and gynecology department of different hospitals and peripheral clinics in Lebanon	CS	Consecutive	Pregnant women, at 35–37 weeks of gestation	All	Self-reported or reported by physician	107	7	6.5
Khalil MM and Alzahra E (84)	1/2009–12/2010	Libya, Tripoli	Al-Jalaa Maternity Hospital	CS	Consecutive	Pregnant women with singleton pregnancies who completed 28 weeks of gestation excluding stillbirths, neonatal deaths, and infants with congenital anomalies	All	Medical records	28,140	405	1.4
Utz B et al. (85)	12/2016–03/2017	Morocco, Marrakech-Safi	10 health centers per district; two districts, Marrakech and Al Haouz	CS	Whole population	Pregnant women attending ANC with GDM screening and management intervention	All	WHO 2013	846	155	18.3
						Pregnant women attending ANC with GDM screening and initial management			1034	138	13.4
Abdwani R et al. (86)	01/2007–12/2013	Oman, Seeb	Sultan Qaboos University Hospital	RS	Consecutive	Mothers with systemic lupus erythematosus	All	Medical Records	56	15	26.8
						Healthy mothers			91	9	9.9
Al-Hakmani FM et al. (87)	3/2011–4/2012	Oman, Seeb	All primary health care centers	PS	Consecutive	Pregnant women without preexisting diabetes or chronic disease tested in their second trimester	All	WHO 1999	638	100	15.7
							BMI: 18.5–24.9 kg/m^2^		229	27	11.8
							BMI: 25–29.9 kg/m^2^		197	35	17.8
							BMI: ≥30 kg/m^2^		212	38	17.9
Abu-Heija AT et al. (88)	09/15/2013–09/14/2014	Oman, Muscat	Sultan Qaboos University Hospital	CS	Whole population	Healthy singleton Omani nondiabetic pregnant women attending the antenatal clinic at SQUH were studied	All	Unclear	306	23	7.5
							BMI: 18–20 kg/m^2^		32	1	3.1
							BMI: 21–25 Kg/m^2^		74	3	4.1
							BMI: 26–30 kg/m^2^		102	8	7.8
							BMI: 31–35 kg/m^2^		47	5	10.6
							BMI: >35 kg/m^2^		51	6	11.8
Zutshi A et al. (89)	11/2011–04/2012	Oman, Muscat	Royal Hospital in Muscat	RC	Whole population	All pregnant Omani women with available weight/height or BMI data at <12 gestational weeks (obese and normal weight)	All	Medical records	1813	221	12.2
							Normal weight		912	69	7.6
							Obese		901	152	16.9
Islam M et al. (90)	2000–2000	Oman	National Health household survey	CS	Multistage sampling	15–49-year-old pregnant women	All	Self–reported	1,345	44	3.3
							20–34 years		1,030	30	2.9
							≥35 years		315	14	4.4
Al–Kuwari MG et al. (91)	1/3–30/6/2010	Qatar	Sixteen primary health care centers that offer ANC care services	CS	Unclear	All pregnant women attending ANC clinics with a mean age of 28.3 years	All	ADA 2003	4,295	275	6.4
							<24 years		1,140	27	2.4
							25–29 years		1,537	89	5.8
							30–34 years		1,007	70	7.0
							≥35 years		611	89	14.6
Bener A et al. (92)	1/2010–4/2011	Qatar	Women’s Hospital in Doha	CS	Whole population	All pregnant women who attended the ANC clinics, excluding women with diabetes before pregnancy	All	Unclear	1,608	262	16.3
							BMI: <25 kg/m^2^		513	35	6.8
							BMI: 25–30 kg/m^2^		601	72	12.0
							BMI: >30 kg/m^2^		494	155	31.4
Abu Yaacob S et al. (93)	01/2001–06/2001	Doha, Qatar	Women’s Hospital	CS	Random	Postnatal women at the Women’s Hospital; multiple pregnancies were not included	All	Medical records	150	35	23.3
							BMI: >30 kg/m^2^		75	26	34.7
							BMI: 20–28 kg/m^2^		75	9	12.0
Bashir M et al. (94)	03/2015–12/2016	Qatar, Doha	Women’s Hospital of Hamad Medical Corporation	CS	Whole population	Pregnant women	All	Medical records, FBG at first trimester and OGTT at second trimester according to WHO	2,221	801	36.1
Shaukat S and Nur U (95)	06/01/2016–11/10/2017	Qatar	Primary Healthcare Corporation Database	RC	Whole population	Nulliparous women with singleton pregnancies who had their first antenatal visit at the Primary Healthcare Corporation	All	Medical records	1,134	407	35.9
							BMI: <25 Kg/m^2^		404	118	29.2
							BMI: 25–29.99 Kg/m^2^		399	140	35.1
							BMI: ≥30 kg/m^2^		230	108	47.00
							Missing		101	41	40.6
Soliman A et al. (96)	01/2017–08/2017	Qatar, All Qatar	Perinatal registry	CS	Whole population	Women with singleton births and completed record abstraction	All	IADPSG	12,255	3027	24.7
							≤19 years		256	35	13.7
							20–24 years		2,075	332	16.0
							25–29 years		4,035	909	22.5
							30–34 years		3,641	964	26.7
							≥35 years		2,275	787	34.6
Kurdi AM et al. (97)	07/01/2010–06/30/2013	Saudi Arabia, Riyadh	The Prince Sultan Military Medical City (PSMMC) is a tertiary teaching institution	PC	Random	Healthy pregnant women	All	IADPSG	1262	188	14.9
					Whole population	Pregnant women with congenital anomalies	All		1179	187	15.9
El–Gilany AH and Hammad S (98)	2007	Saudi Arabia, Al–Hassa	Primary health care centers	PS	Unclear	Pregnant women initiated into ANC in the first month of pregnancy, excluding any prepregnancy chronic medical disease (e.g., hypertension, diabetes, renal or cardiac disease, and sickle cell disease) and multiple pregnancies	All	Unclear	787	30	3.8
							BMI: 18.5–24.99 kg/m^2^		307	3	1.0
							BMI: <18 kg/m^2^		67	0	0.0
							BMI: ≥25–29.99 kg/m^2^		187	8	4.3
							BMI: ≥30 kg/m^2^		226	19	8.4
Lasheen AE et al. (99)	1/2011–11/2011	Saudi Arabia, Riyadh	Security Forces Hospital	CS	Unclear	Pregnant women	All	Unclear	601	153	25.5
Wahabi HA et al. (100)	2013–2015	Saudi Arabia, Riyadh	Three hospitals, part of RAHMA study	CS	Random	Saudi mothers	All <20–≥45 years	WHO 2013
									9,723	345	3.5
							<20 years		216	38	17.6
							20–24 years		1,625	271	16.7
							25–29 years		2,850	596	20.9
							30–34 years		2,603	688	26.4
							35–39 years		1,769	537	30.4
							40–44 years		601	208	34.6
							≥45 years		59	16	27.1
Wahabi HA et al. (101)	1/1/–31/12/2008	Saudi Arabia, Riyadh	King Khalid University Hospital	RS	Unclear	Women who were admitted to the labor ward in King Khalid University Hospital	All	IADPSG	3,157	569	18.0
Wahabi HA et al. (102)	1/1–31/12/2010	Saudi Arabia, Riyadh	King Khalid University Hospital	RS	Unclear	Pregnant women with singleton pregnancies at gestational age of at least 24 months excluding women with preexisting diabetes	All	IADPSG	3,041	569	18.7
Wahabi HA et al. (103)	1/7/2011–30/6/2012	Saudi Arabia, Riyadh	King Khalid University hospital	RS	All subjects during the study period	Women booked for ANC care services who were with singleton pregnancies and with no history of T1DM or T2DM	All	Carpenter and Coustan	2,701	415	15.4
							Obese		1,185	260	21.9
							Not obese		1,516	155	10.2
Al-Rowaily MA and Abolfotouh MA (104)	7/2005–7/2006	Saudi Arabia, Riyadh	ANC clinic of King Fahd hospital, part of the National Guard Health Affairs services	CS	Consecutive	All pregnant women who had no previous history of diabetes without pregnancy excluding women who suffered an abortion before reaching 24–28 weeks gestation; 50.1% of pregnant women were grand multiparas	All	WHO 1985	633	79	12.5
							<20 years		21	0	0.0
							20–29 years		180	10	5.6
							30–39 years		379	54	14.2
							≥40 years		53	15	28.3
Almarzouki AA (105)	1/11/2007–30/4/2008	Saudi Arabia, Makkah	Department of endocrinology, Al-Noor Specialist Hospital	RS	All pregnant women during the study period	All singleton pregnant women excluding pregnant women known to have DM before pregnancy or who have OGTT positive in first trimester of pregnancy with unknown prepregnancy DM status were also excluded	All	O’Sullivan and NDDG	1,550	94	6.1
Al–Shaikh G et al. (106)	2014–2014	Saudi Arabia, Riyadh	Labour ward of King Khaled University Hospital	CS	Consecutive	17–47-year-old pregnant women who were admitted for delivery	All	Unclear	1,000	111	11.1
Al-Daghri N et al. (107)	Unclear	Saudi Arabia, Riyadh	Patients recruited from homes and invited to visit primary healthcare centers.	CS	Random	18–45-year-old pregnant women attending clinics	All	WHO 1999	2,373	33	1.4
Wahabi H et al. (108)	2013–2015	Saudi Arabia, Riyadh	Large tertiary care public hospitals	CS	Whole population	Women delivered at participating hospitals with a mean age of 29.1 years	<20–≥40 years	WHO 2013	9,723	2,354	24.2
Alfadhli E et al. (109)	2011–2014	Saudi Arabia, Medina	Maternity and Children hospital	PC	Consecutive	Singleton Saudi pregnant women without DM and with mean age 30.5 years	All	ADA 2010	573	93	16.2
Al Serehi A et al. (110)	2011–2013	Saudi Arabia, Riyadh	Single-center study conducted at King Fahad Medical City	CS	Whole population	Pregnant women with a mean age of 29.9 years; trimester not mentioned	All	Medical records	1,718	238	13.8
Al–Rubeaan K et al. (111)	2007–2009	Saudi Arabia, Nationwide	SAUDI–DM national level household survey.	CS	Random	Pregnant women in different trimesters, recruited from general population with an age range of 18–49 years	All 18–49 years	IADPSG criteria
									549	201	36.6
							18–29 years		264	79	29.9
							30–39 years		212	85	40.1
							40–49 years		73	37	50.7
Gasim T et al. (112)	2001–2008	Saudi Arabia	King Fahad Hospital	CC	Matched random sampling	Pregnant women in their second trimester with a mean age of 32.4 years	All	IADPSG	8,075	220	2.7
Kurdi MA et al. (113)	01/2000–12/2001	Saudi Arabia, Riyadh	Armed Forces Hospital and King Khalid University Hospital	CS	Consecutive	Pregnant women with multiple pregnancies	All	Unclear	375	60	16.0
Abdelmola AO et al. (114)	11/2014	Saudi Arabia, Jazan	Sabya, Jazan, and Abuarish hospitals	CS	Random	Pregnant women aged 15–49 years in the second and third trimester tested for GDM at 24–28 weeks	15–20 years	Medical records	48	6	12.5
							21–25 years		145	3	2.1
							26–30 years		136	13	9.6
							31–35 years		76	10	13.2
							36–50 years		35	4	11.4
Al-Shaikh GK et al. (115)	11/2013- 11/2014	Saudi Arabia, Riyadh	King Khaled University Hospital	CS	Whole population	Women who had singleton births	All	Medical records	3,327	415	12.5
							Primipara		1,889	174	9.3
							Multipara		1,097	156	14.4
							Grand multipara		341	85	25.2
Fayed AA et al. (116)	11/2013–03/2015	Saudi Arabia, Riyadh	Multicenter Mother and Child Cohort Study RAHMA, three hospitals in Riyadh	CS	Systematic	RAHMA study recruited more than 14,000 pregnant women and their newborns from three hospitals representing the ministry of health, military and university hospitals; all Saudi women were eligible to participate, and 14,568 consented	All 15–39 years	WHO 2013
									9,022	2,124	23.5
							15–20 years		181	32	17.7
							20–29 years		4,469	867	19.4
							30–34 years		2,606	688	26.4
							35–39 years		1,766	537	30.4
Subki AH et al. (117)	01/2015–06/2017	Saudi Arabia, Jeddah	King Abdulaziz University Hospital, a teaching hospital and tertiary health center located in the city of Jeddah in the western province of Saudi Arabia	CS	Whole population	All patients diagnosed with HDP	All	Medical records	244	59	26.3
							Primigravida		97	18	18.6
							Multigravida		127	41	32.3
Al Shanqeeti SA et al. (118)	01/2016–08/2016	Saudi Arabia, Riyadh	King Abdulaziz Medical City	CS	Whole population	Pregnant women attending the antenatal clinic at the tertiary hospital as well as those admitted for OB/GYN care and women attending the antenatal clinic at the primary care center were invited to participate in this study	All	Unclear	384	35	9.1
Dafa Elseed EB and Khougali HS (119)	01/01/2016–06/01/2017	Sudan Omdurman	Outpatient clinical at Omdurman Maternity Hospital, Omdurman, Sudan	CS	Unclear	Women with diabetes aged 18–45 years	All	Self-reported	119	55	46.2
Naser W et al. (120)	01/2015–11/2015	Sudan, Khartoum	ANC clinic of Saad Abualila Hospital	PC	Whole population	Singleton pregnant, started ANC follow-up in the first trimester (≤14 weeks of gestation)	All	IADPSG and ADA	126	19	15.0
Alshareef SA et al. (121)	07/01/2017–01/31/2018	Sudan, Khartoum	Saad Abuelela hospital	CS	Unclear	Pregnant women	All	IADPSG	166	20	12.0
Mallouli M et al. (122)	01/01–31/12, 2013	Tunisia, Sfax	University Hospital, HediChaker	CS	Whole population	Mothers of macrosomic newborn	All	ADA 2015	821	76	9.3
Radwan H et al. (123)	6/2016	UAE, Sharjah, Dubai and Ajman	Three main public governmental hospitals and seven rimary health care (PHC) clinics and mother and child centers (MCH)	PC	Convenient	Singleton Arab aged 19–40 years within the third trimester of pregnancy (27–42 weeks of gestation)	All	NICE	256	49	19.2
Agarwal MM et al. (124)	1/1998–12/2002	UAE, Al Ain	Obstetric clinics at the Al Ain Hospital	RS	Unclear	Pregnant women attending routine obstetric clinics at the Al Ain Hospital with a mean maternal age of 32 years	All	ADA 1997	5,347	1,641	30.7
Agarwal MM et al. (125)	1/1/2012–31/12/2012	UAE, Al Ain	Tawam Hospital	CS	Unclear	Pregnant women attending the routine ANC clinics	All	ADA 2003	2,337	310	13.2
Agarwal MM et al. (126)	2003–2008	UAE, Al Ain	Antenatal clinics of two tertiary care hospitals	PC	Whole population	Pregnant women attending antenatal clinics	All	ADA 2010	10,283	1328	12.9
Agarwal MM et al. (127)	1/07/2007–30/06/2008	UAE, Al Ain	Al Ain Hospital	CS	Unclear	Pregnant women attending routine antenatal clinics tested for GDM at 24–28 weeks’ gestation	All	ADA 2007	1,465	196	13.4
Mirghani MH et al. (128)	01/2002–05/2004	UAE, Al Ain	Al-Ain Hospital, Al Ain District	CS	Consecutive	Healthy pregnant women fasting in the month of Ramadan	All	WHO 1999	168	34	20.2
						Healthy pregnant women not fasting in the month of Ramadan			156	11	7.1
Agarwal MM et al. (129)	1/5/2003–31/7/2003	UAE, Al Ain	Tawam Hospital, Al Ain	CS	Consecutive	All pregnant women undergoing one-step universal screening protocol for GDM between 24–28 weeks gestation	All	ADA 2004	442	49	11.1
Vaswani PR et al. (130)	12/2010–10/2011	UAE, Abu Dhabi	Mafraq hospital	CS	Consecutive	Pregnant women except the ones with multiple pregnancies or BMI less than 18.5 kg/m^2^ or preexisting hypertension or diabetes	All	Medical records	1,985	171	8.6
							Overweight		635	36	5.6
							Obese class I		520	53	10.1
							Obese class II		280	42	1.0
							Obese class III		130	23	17.6
							Normal weight		420	17	4.0
Abdel–Wareth OL et al. (131)	11/1999–04/2001	UAE, Abu Dhabi	Mafraq Hospital	CS	Consecutive	Women delivering at Mafraq Hospital during the time period were included; women who could not perform the test due to vomiting were excluded from the study	<25–≥35 years	ADA criteria	877	143	16.3
Ali AD. et al. (132)	08/2013–03/2014	Yemen, Dhamar	Antenatal care clinics associated with several hospitals	CS	Systematic	Pregnant women visiting antenatal clinics with a mean age of 25.1 years	Obese	ADA criteria	18	3	16.7
							Others		293	13	4.4

ACOG, American College of Obstetricians and Gynecologists; ADA, American Diabetes Association; ANC, antenatal care; ART, assisted reproductive technology; BMI, body mass index; CC, case control; CS, cross-sectional; DM, diabetes mellitus; FIGO, Federation of Gynecology and Obstetrics; GDM, gestational diabetes mellitus; HCV, hepatitis C virus; HDP, hypertension disorder in pregnancy; IADPSG, International Association of Diabetes and Pregnancy Study Groups; ICSI, intracytoplasmic sperm injection; IVF, in vitro fertilization; NDDG, National Diabetes Data Group; OGTT, oral glucose tolerance test; PC, prospective cohort; PCOS, polycystic ovary syndrome; PS, prospective; PTB, preterm birth; RC, retrospective cohort; RS, retrospective; SC, spontaneous conception; T1DM, type 1 diabetes mellitus; T2DM, type 2 diabetes mellitus; WHO, World Health Organization.

### Crude GDM Prevalence

The 102 research reports (31–67, 69–132) yielded 198 GDM prevalence studies. Iran (32.3%) (41, 43–67, 69–77) and Saudi Arabia (24.2%) (97–118) contributed to most of the prevalence studies, followed by Qatar (9.7%). In these prevalence studies, a total of 279,202 pregnant women were tested for GDM between 2000 and 2019, and the crude GDM prevalence was estimated to be about 11.0%. The prevalence of GDM ranged from 0.0% in three studies (60, 98, 104) to 50.7% in pregnant women aged 40–49 years in Saudi Arabia tested between 2007 and 2009 (111). The GDM prevalence range was identical in studies reported in the two decades (**Tables 2** and **3**).

**Table 3:** Weighted national prevalence of GDM in pregnant women in 16 MENA countries by study period and overall.

Country/study period	No. of studies	Tested sample	GDM	GDM prevalence				Heterogeneity measures			*p*–value^4^ (fixed model)
				Range (%)	Median (%)	Weighted prevalence %	95% CI	Q (*p*–value)^1^	*I^2^* (%)^2^	95% prediction interval (%)^3^	
**Algeria**											—
2010–2019	1	200	6	—	—	3.0	1.4–6.4	—	—	—	
**Bahrain**											<0.001 (<0.001)
2000–2009	2	10,495	1,394	7.5–15.5	11.5	13.0	12.4–13.7	—	—	—	
2010–2019	9	49,552	4,982	6.9–13.3	9.5	9.7	8.1–11.6	352.4 (*p*<0.001)	97.7	4.2 – 17.2	
Overall	11	60,047	6,376	6.9–15.5	9.5	10.0	8.3–11.9	572.3 (*p*<0.001)	98.3	4.0–18.3	
**Egypt**											0.21 (0.002)
2010–2019	4	1,239	184	5.9–26.3	11.2	13.5	6.2–21.8	49.9 (*p*<0.001)	94.0	0.0–63.8	
Overlapping	2	308	24	7.6–7.9	7.8	7.8	5.0–11.1	—	—	—	
Overall	6	1,547	208	5.9–26.3	9.0	11.2	6.2–17.4	59.7 (*p*<0.001)	91.6	0.0–37.7	
**Iran**											0.07 (<0.001)
2000–2009	16	7,343	492	2.2–24.4	7.4	8.2	5.9–11.0	215.3 (*p*<0.001)	93.0	0.8–21.9	
2010–2019	39	21,028	2,235	0.0–50.0	9.2	12.3	9.0–16.0	2,135 (*p*<0.001)	98.2	0.0–41.0	
Overlapping	9	1,388	166	5.9–28.6	13.5	13.5	8.2–19.7	67.8 (*p*<0.001)	88.2	0.3–38.4	
Overall	64	29,759	2,893	0.0–50.0	8.8	11.4	9.2–13.9	2,491 (*p*<0.001)	97.5	0.1–35.8	
**Iraq**											—
2010–2019	4	383	35	2.6–24.5	14.2	11.5	3.3–23.3	24.5 (*p*<0.001)	87.8	0.0–76.6	
**Jordan**											—
2010–2019	6	43,847	604	1.2–31.7	4.7	4.7	3.0–6.7	193.7 (*p*<0.001)	97.4	0.4–12.5	
**Lebanon**											—
2010–2019	2	211	23	6.5–15.4	11.0	10.5	6.7–15.1	—	—	—	
**Libya**											—
Overlapping	1	28,140	405	–	–	1.4	1.3–1.6	—	—	—	
**Morocco**											—
2010–2019	2	1,880	393	13.3–18.3	15.8	15.5	13.9–17.2	—	—	—	
**Oman**											<0.001 (<0.001)
2000–2009	2	1,345	44	2.9–4.4	3.7	3.2	2.3–4.2	—	—	—	
2010–2019	10	2,757	344	3.1–17.9	11.2	11	8.0–15.0	59.2 (*p*<0.001)	84.8	1.9–25.8	
Overlapping	2	147	24	9.9–26.8	18.3	15.5	10–21.9	–	–	–	
Overall	14	4,249	412	2.9–26.8	10.3	10.1	6.5–14.3	184.5 (*p*<0.001)	93.0	0.2–29.7	
**Qatar**											0.65 (0.59)
2000–2009	2	150	35	12.0–34.7	23.3	22.3	15.9–29.4	–	–	–	
2010–2019	17	21,513	4,772	2.4–47.0	22.5	20.5	14.8–26.9	1,869.0 (*p*<0.001)	99.1	1.6–52.6	
Overall	19	21,663	4,807	2.4–47.0	22.5	20.7	15.2–26.7	1,880.3 (*p*<0.001)	99.0	1.7–52.4	
**Saudi Arabia**											0.02 (<0.001)
2000–2009	16	17,499	1,286	0.0–50.7	7.2	10.8	6.2–16.5	1,330.5 (*p*<0.001)	98.9	0.0–41.1	
2010–2019	32	44,918	9,331	2.1–34.6	17.6	18.2	15.9–20.6	1,116.5 (*p*<0.001)	97.2	7.1–32.9	
Overall	48	62,417	10,617	0.0–50.7	16.1	15.5	12.6–18.8	4,989.3 (*p*<0.001)	99.1	1.0–41.9	
**Sudan**											—
2010–2019	3	411	94	12.0–46.2	15.1	23.0	3.3–45.2	47.2 (*p*<0.001)	95.8	—	
**Tunisia**											—
2010–2019	1	821	76	—	—	9.3	7.5–11.4	—	—	—	
**United Arab Emirates**											0.3 (<0.001)
2000–2009	7	18,738	3,402	7.1–30.7	13.4	15.5	9.2–23.0	736.7 (*p*<0.001)	99.2	0.2–46.9	
2010–2019	7	4,578	530	4.0–19.1	13.3	11.3	7.6–15.69	87.8 (*p*<0.001)	93.2	1.3–28.8	
Overall	14	23,316	3,932	4.0–30.7	13.3	13.4	9.4–18.0	945.1 (*p*<0.001)	98.6	1.1–35.6	
**Yemen**											
2010–2019	2	311	16	—	—	—	—	—	—	—	—
**Overall^5^**	**198**	**279,202**	**30,797**	**0.0–50.7**	**12.3**	**13.0**	**11.5–14.6**	**28,154 (p<0.001)**	**99.3**	**0.1–40.6**	—

CI, confidence interval calculated using the exact binomial method; GDM, gestational diabetes mellitus; MENA, Middle East and North Africa.

^1^Q: Cochran’s Q statistic is a measure assessing the existence of heterogeneity in estimates of GDM prevalence.

^2^I^2^ is a measure assessing the percentage of between-study variation due to differences in GDM prevalence estimates across studies rather than chance.

^3^Prediction intervals estimate the 95% confidence interval in which the true GDM prevalence estimate in a new study is expected to fall.

^4^Heterogeneity between subgroups using random-effects model (fixed-effect model).

^5^Overall pooled estimates in the 16 countries regardless of the tested population, sample size, and data collection period, using the most updated criteria when GDM is ascertained using different criteria in the same population.

### Regional and National Pooled GDM Prevalence

**Figure 2:**
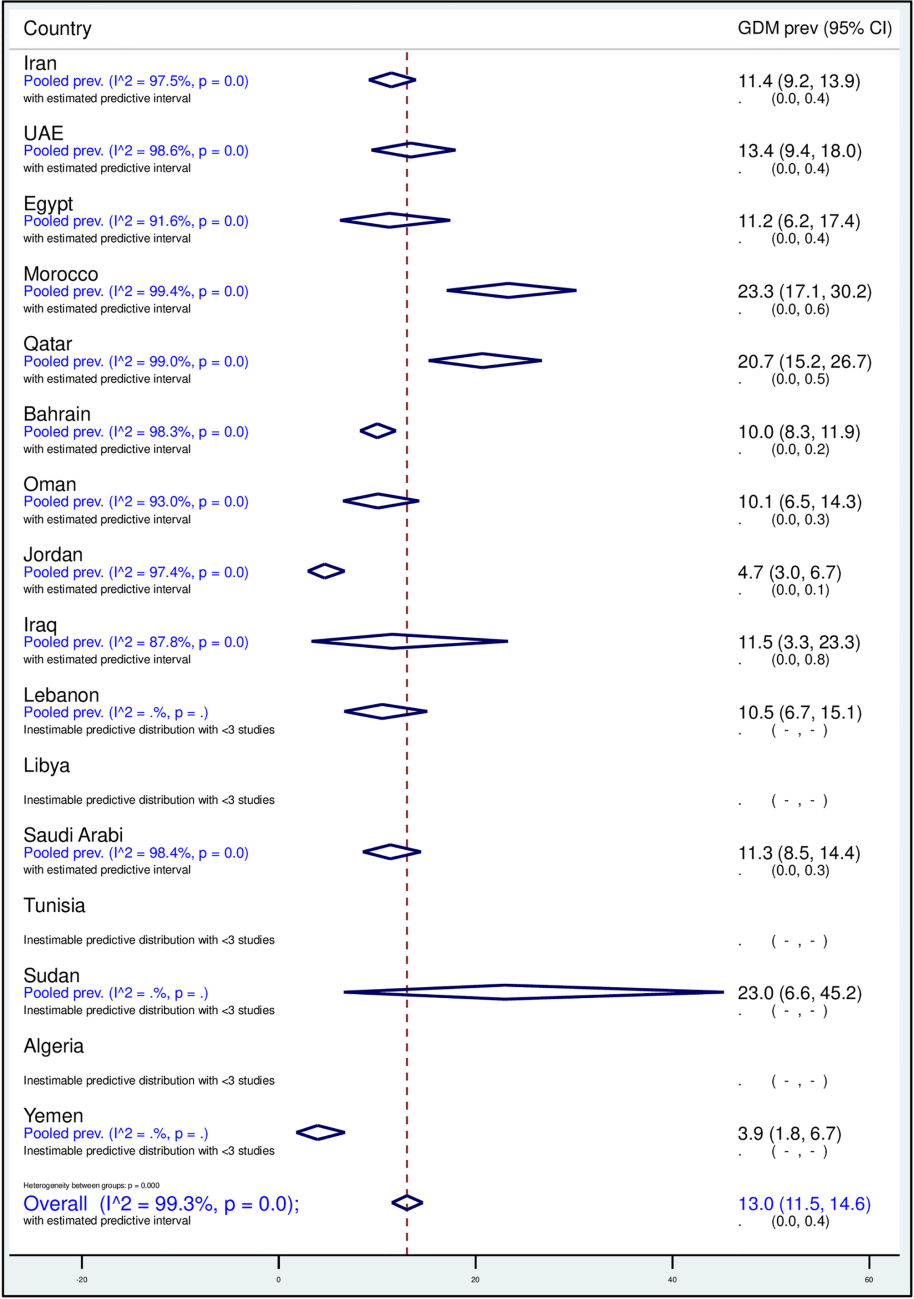
Forest plot of the meta-analyses of the studies on GDM from 16 MENA countries.

The overall pooled weighted GDM prevalence in the MENA region was 13.0% (95% CI, 11.5–14.6%, *I^2^*, 99.3%; **Table 3**; **Figure 2**). The highest GDM prevalence was observed in Qatar (20.7%, 95% CI, 15.2–26.7%; 19 studies), followed by 15.5% in Saudi Arabia (95% CI, 12.6–18.8%; 48 studies) and 13.4% in the UAE (95% CI, 9.4–18.0%; 14 studies; **Table 3**). The lowest pooled GDM prevalence was 4.7% in Jordan (95% CI, 3.0–6.7%; six studies) reported between 2010 and 2019. In the studies conducted between 2000 and 2009, the prevalence estimates ranged from 3.2% in Oman (95% CI, 2.3–4.2%) to 22.3% in Qatar (95% CI, 15.9–29.4%), and in the studies conducted between 2010 and 2019, it ranged from 3.0% in Algeria (95% CI, 1.4–6.4%) to 23.0% in Sudan (95% CI, 3.3–45.2%; **Table 3**).

For the six countries reporting data on both decades, the overall GDM prevalence was estimated separately for each decade. There was a rise in the prevalence of GDM by 4% to 8% in Iran, Oman, and Saudi Arabia and a decrease of 2% to 4% in Bahrain, Qatar, and the UAE from 2000–2009 to 2010–2019 periods. The largest increase in prevalence occurred in Oman: from 3.2% in 2000 (95% CI, 2.3–4.2%) to 11.0% in 2019 (95% CI, 8.0–15.0%, *I^2^*, 84.2%). An appreciable reduction in the prevalence of GDM was observed in the UAE: from 15.5% in 2000 (95% CI, 9.2–23.0%, *I^2^*, 99.2%) to 11.3% in 2019 (95% CI, 7.6–15.69, *I^2^*, 93.2%; **Tables 2** and **3**).

### Subgroup Pooled GDM Prevalence

The prevalence of GDM in pregnant women aged ≥30 years was 2.26 times higher (21.9%, 95% CI, 18.5–25.5%, *I^2^*, 97.1%) than that estimated in younger (15–29 years) pregnant women (9.7%, 95% CI, 6.7–13.2%, *I^2^*, 98.0%). A trend was observed between GDM and pregnancy trimester. The weighted GDM prevalence increased by 45.0%, from 8.9% in the first trimester to 12.9% in the second trimester, and by 55.0% in the third trimester (20.0%, 95% CI, 13.1–27.9%, *I^2^*, 98.8%) compared with the second trimester. It was also noticeable that, as the BMI increased, the prevalence of GDM increased by 54% in overweight (12.0%, 95% CI, 5.7–20.1%, *I^2^*, 96.7) and by 120% in obese (17.2%, 95% CI, 12.8–22.0%, *I^2^*, 93.8%) compared with normal-weight pregnant women (7.8%, 95% CI, 4.1–12.4%, *I^2^*, 95.0%). No GDM cases were reported in two studies that included underweight women (**Table 4**).

**Table 4:** Subgroup weighted prevalence of GDM in pregnant women in 16 MENA countries by age, pregnancy trimester, body mass index, study period, ascertainment methodology, tested sample, C-section, and maternal mortality ratio.

	No. of studies	Tested sample	GDM	GDM prevalence				Heterogeneity measures			*p*–value^4^(fixed model)
				Range (%)	Median (%)	Weighted prevalence %	95% CI	Q (*p*–value)^1^	*I^2^*(%)^2^	95% prediction interval (%)^3^	
**Age**											<0.001 (<0.001)
15–29 years	24	19,187	2,883	0.0–29.9	10.8	9.7	6.7–13.2	1,140.7 (p<0.001)	98.0	0.0–31.4	
≥30 years	26	22,186	5,617	4.1–50.7	25.4	21.9	18.5–25.5	868.6 (p<0.001)	97.1	7.0–42.0	
Unclear age	148	237,518	22,281	0.0–50.0	11.2	12.3	10.6–14.0	20,967.2 (p<0.001)	99.3	0.1–37.6	
**Trimester**											0.06 (<0.001)
First	11	5,807	387	2.2–37.2	7.6	8.9	5.3–13.3	272.5 (p<0.001)	96.3	0.0–29.7	
Second	85	134,792	14,378	0.0–50.0	12.0	12.9	10.9–15.0	9,687.2 (p<0.001)	99.1	0.6–36.3	
Third	18	14,146	1,354	2.7–50.7	18.5	20.0	13.1–27.9	1,428.2 (p<0.001)	98.8	0.0–60.6	
Not reported	84	124,457	14,678	0.0–47.0	12.5	12.5	9.8–15.5	16,618.8 (p<0.001)	99.5	0.0–46.1	
**BMI**											<0.001 (<0.001)
Underweight	2	94	0	0	0	0	—	—	—	—	
Normal weight	11	3,822	335	1.0–29.2	6.0	7.8	4.1–12.4	200.8 (p<0.001)	95.0	0.0–29.5	
Overweight	7	2,502	334	4.3–35.1	9.2	12.0	5.7–20.1	182.2 (p<0.001)	96.7	0.0–47.5	
Obese	17	4,8459	941	7.6–47.0	15.8	17.2	12.8–22.0	241.5 (p<0.001)	93.8	2.6–40.2	
Unclear	161	267,6925	29,187	0.0–50.7	12.8	13.4	11.7–15.2	27,066.0 (p<0.001)	99.4	0.1–41.2	
**Study period**											0.14 (<0.001)
2000–2009	45	55,570	6,653	0.0–50.7	11.1	10.6	8.1–13.4	4,118.0 (p<0.001)	98.9	0.0–34.2	
2010–2019	139	193,3649	23,527	0.0–50.0	12.7	14.0	12.1–16.0	19,613.9 (p<0.001)	99.3	0.2–42.2	
Overlapping	14	29,983	619	1.4–28.6	9.1	12.0	6.5–18.7	414.1 (p<0.001)	96.9	0.0–45.3	
**GDM ascertainment** ^5^											<0.001 (<0.001)
WHO guidelines											
WHO 1985	4	633	79	0.0–28.3	9.9	10.4	3.2–20.5	25.4 (p<0.001)	88.2	0.0–67.5	
WHO 1999	6	3,335	178	1.4–20.2	14.8	11.4	3.6–22.8	228.9 (p<0.001)	97.8	0.0–62.4	
WHO 2013	14	30,348	7,125	13.3–34.6	22.6	22.8	20.2–25.5	344.5 (p<0.001)	96.2	13.0–34.5	
WHO year not mentioned	1	2,221	801	—	—	36.1	34.1–38.1	—	—	—	
ADA guidelines				—	—			—	—	—	
ADA 1997	1	5,347	1,641	—	—	30.7	29.5–31.9	—	—	—	
ADA 2002–2010	16	19,604	2,269	2.4–37.2	12.0	11.7	9.0–14.7	364.6 (p<0.001)	96.4	2.6–25.9	
ADA 2011–2013	4	3,180	605	13.4–30.5	24.9	22.7	15.4–30.9	67.5 (p<0.001)	95.6	0.2–65.0	
ADA 2015–2016	4	3,229	243	6.9–9.3	7.0	7.5	6.4–8.7	4.435 (p=0.218)	32.4	4.2–11.7	
ADA year not mentioned	1	877	143	—	—	16.3	14.0–18.9	—	—	—	
ADA/IADPSG	2	700	306	15.1–50.0	32.5	43.1	39.4–46.8	—	—	—	
Self-reported	6	1,833	119	2.9–46.2	5.5	9.6	2.7–19.8	148.2 (p<0.001)	96.6	0.0–56.2	
Medical records	45	70,833	2,803	0.6–47.0	11.4	11.5	9.1–14.2	3,588.1 (p<0.001)	98.8	0.4–33.1	
Unclear	36	31,541	1,319	0.0–31.4	8.4	9.3	6.2–12.9	1,770.5 (p<0.001)	98.0	0.0–36.9	
IADPSG	23	32,911	5,577	2.7–50.7	18.0	20.9	15.6–26.6	3,071.8 (p<0.001)	99.3	1.5–53.5	
Carpenter and Coustan	13	8,468	755	2.2–24.4	8.2	8.8	5.6–12.7	356.1 (p<0.001)	96.6	0.1–27.4	
NDDG	10	51,102	5,076	6.1–13.3	8.7	9.4	7.8–11.1	382.7 (p<0.001)	97.6	4.0–16.7	
Fourth International Workshop–Conference	2	10,495	1,394	7.5–15.5	11.5	13.0	12.4–13.7	—	—	—	
Fifth International Workshop–Conference	5	1,010	215	7.3–44.4	7.9	17.4	5.6–33.9	149.9 (p<0.001)	97.3	0.0–85.6	
ACOG	4	1,279	100	0.0–16.7	7.6	7.7	3.7–12.9	18.11 (p<0.001)	83.4	0.0–36.8	
NICE	1	256	49	—	—	19.1	14.8–24.4	—	—	—	
**Sample size**											0.25 (<0.001)
<100	32	1,779	300	0.0–50.7	12.8	14.8	10.7–19.5	198.8 (p<0.001)	84.4	0.0–44.3	
≥100	166	277,423	30,497	0.6–50.0	12.0	12.8	11.2–14.5	27,873.7 (p<0.001)	99.4	0.1–40.1	
**C-section rate**											<0.001 (<0.001)
<15%	7	10,206	481	2.7–46.2	12.0	11.5	5.6–19.0	285.6 (p<0.001)	97.9	0.0–44.2	
15–29%	118	235,106	27,222	0.0–50.7	13.5	14.4	12.3–16.6	24,307.1 (p<0.001)	99.5	0.2–43.3	
>30%	69	29,101	3,010	0.0–50.0	9.2	11.6	9.4–14.1	2,461.8 (p<0.001)	97.2	0.1–36.1	
Unclear	4	4,789	147	1.4–15.0	3.9	4.8	1.8–9.0	89.2 (p<0.001)	96.6	0.0–34.3	
**Maternal mortality ratio**											<0.001 (<0.001)
≤100/100,000	188	273,491	30,534	0.0–50.7	12.5	13.2	11.6–14.9	27,551.7 (p<0.001)	99.3	0.1–40.8	
>100/100,000	6	922	1116	3.0–46.2	13.6	16.5	3.4–36.3	97.1 (p<0.001)	96.9	0.0–100.0	
Unclear	4	4,789	147	1.4–15.0	3.9	4.8	1.8–9.0	89.2 (p<0.001)	96.6	0.0–34.3	
** Overall^6^**	**198**	**279,202**	**30,797**	**0.0–50.7**	**12.3**	**13.0**	**11.5–14.6**	**28154 (p<0.001)**	**99.3**	**0.1–40.6**	—

CI, confidence interval calculated using the exact binomial method; ACOG, American College of Obstetricians and Gynecologists; ADA, American Diabetes Association; GDM, gestational diabetes mellitus; IADPSG, International Association of Diabetes and Pregnancy Study Groups; NDDG, National Diabetes Data Group; NICE, National Institute for Health and Care Excellence; WHO: World Health Organization.

^1^Q: Cochran’s Q statistic is a measure assessing the existence of heterogeneity in estimates of GDM prevalence.

^2^I^2^ is a measure assessing the percentage of between-study variation due to differences in GDM prevalence estimates across studies rather than chance.

^3^Prediction intervals estimate the 95% confidence interval in which the true GDM prevalence estimate in a new study is expected to fall.

^4^Heterogeneity between subgroups using random-effects model (fixed-effect model).

^5^Regardless of the year of the guidelines for the most updated criteria when GDM was ascertained, based on different criteria in the same population.

^6^Overall pooled estimates in the 16 countries regardless of the tested population, sample size, and data collection period, using the most updated criteria when GDM was ascertained using different criteria in the same population.

From the 137 studies conducted between 2010 and 2019, the pooled GDM prevalence (14.0%, 95% CI, 12.1–16.0%) was 32.0% higher than that reported in the 45 studies conducted in the previous decade (2000–2009; 10.6%, 95% CI, 8.1–13.4%). The pooled GDM prevalence was relatively higher in 32 studies with a sample size of <100 pregnant women (14.8%, 95% CI, 10.7–19.5%) compared with that in 164 studies with a sample size of ≥100 pregnant women (12.8%, 95% CI, 11.2–14.8%; **Table 4**).

The prevalence of GDM was 25.2% higher in countries with a C-section rate of 15–29% (weighted estimate of 14.4%, 95% CI, 12.3–16.6%, *I^2^*, 99.5%) than countries with a C-section rate of <15% (weighted estimate of 11.5%, 95% CI, 5.36–19.0%, *I^2^*, 97.9%; **Table 4**). In addition, in four studies in countries with high MMR (i.e., >100 per 100,000 live births), the prevalence of GDM was 25.0% higher than in countries with MMR ≤100 per 100,000 live births (weighted estimates of 16.5%, 95% CI, 3.4–36.3%, and 14.4%, 95% CI, 12.3–16.6%, respectively; **Table 4**).

### Subregional Specific Pooled GDM Prevalence

In Sudan, one of the North African countries with a C-section rate of 15–29%, a lower GDM prevalence (weighted prevalence of 7.9%) was observed compared with countries with a C-section rate of <15% (weighted prevalence of 23.0%). In North African countries with an MMR of >100/100,000 live births, the prevalence of GDM was 32.0% higher than in countries with an MMR of ≤100/100,000 live births (**Supplementary File 2**).

The highest weighted GDM prevalence was in the GCC countries (14.7%, 95% CI, 13.0–16.5%, *I^2^*, 99.0%), followed by North African countries (13.5%, 95% CI, 7.4–20.9%, *I^2^*, 98.9%) and Iran/Iraq 11.2% (95% CI, 9.0–13.5%, *I^2^*, 97.4%), whereas the lowest prevalence was estimated in the Levant region countries (5.8%, 95% CI, 3.9–7.9%, *I^2^*, 97.1%; **Supplementary File 3**).

In GCC countries, the prevalence of GDM rose from 11.9% to 15.9% over the two successive decades. Overweight (12.5%) and obese (18.5%) pregnant women and pregnant women with a C-section rate of 15–29% (15.5%) were burdened with high GDM prevalence (**Supplementary File 3**). In these countries, pregnant women aged ≥30 years were burdened with higher GDM prevalence than the other subregions. As compared with the first decade, the weighted GDM prevalence in the subsequent decade increased by almost 4% in Iraq.

**Tables 2****–4** in the appendix provide additional weighted GDM prevalence estimates in each subregion according to different measured characteristics (**Supplementary Files 2** **–5**).

### Predictors of Heterogeneity in GDM

In the univariate meta-regression models, country, age, pregnancy trimester, BMI, and sample size were associated with variability in the prevalence of GDM at *p*<0.1. In the multivariate meta-regression model, only pregnancy trimester was retained, with no significant association with the prevalence of GDM at *p*<0.05. Compared with Saudi Arabia, the adjusted GDM prevalence was 135% (adjusted odds ratio [aOR], 2.35, 95% CI, 1.39–3.95) and 122% (aOR, 2.22, 95% CI, 1.30–3.76) higher in Qatar and Morocco, respectively, but lower in Libya (aOR, 0.09, 95% CI, 0.02–0.52) and Jordan (aOR, 0.38, 95% CI, 0.18–0.80). Pregnant women aged ≥30 years had a 152% higher prevalence of GDM (aOR, 2.52, 95% CI, 1.51–4.21) relative to younger pregnant women. Obese pregnant women were burdened with a 192% higher prevalence of GDM relative to normal-weight pregnant women (aOR, 2.92, 95% CI, 1.50–5.69; **Supplementary File 6**).

### Publication Bias in GDM Prevalence

Both the visual (funnel plot asymmetry) and statistical assessment (Egger’s test, *p*<0.001) of publication bias suggested the role of a small-study effect (**Supplementary File 7**).

### Quality Assessment of the GDM Research Reports

**Supplementary Figure 2** presents the findings of the research report-specific quality assessment for relevant GDM prevalence studies. In all 102 research reports, the research question(s) and/or objective(s) were clearly stated, and the study population group was clearly specified and defined. Half of the research reports (49.5%) did not provide information on the sample size calculation or justification. Most (79.2%) of the research reports used biological assays or extracted data from medical records to ascertain GDM, whereas the GDM status was self-reported in only five reports. In more than half (58.4%) of the 102 research reports, the tested sample size was at least 100 pregnant women. Overall, the research reports were judged to be of potentially low RoB, with an average of seven of the nine measured assessment items. Four (4.0%) of the reports (70, 85, 105, 120) were of low RoB in all of the assessed RoB items (**Supplementary File 8**).

## Discussion

### Main Findings

A total of 102 eligible research reports comprising 198 GDM prevalence studies were reported in 16 countries in the MENA region between 2000 and 2019. Most of these reports (58.41%) were from Iran and Saudi Arabia. The pooled prevalence of GDM in the 16 MENA countries was appreciably high (13.0%, 95% CI, 11.5–14.6%, *I^2^*, 99.3%), particularly in the GCC and North African countries. The prevalence of GDM increased with maternal age, gestational age, and BMI. It was also high in countries with a C-section rate of 15–29% and an MMR of >100/100,000 live births.

The pooled GDM prevalence (13.0%) was alarmingly higher than that of European countries (2–6%) (133) but was similar to the sub-Saharan Africa region (14.0%). In contrast to the pooled prevalence estimates of Asia (11.5%) (134), the prevalence estimated in the present meta-analysis was slightly higher. The Asian meta-analysis included prevalence estimates from Saudi Arabia, Iran, and Qatar, and when compared with our estimates, they were 3.5% and 7.4% lower for Iran and Saudi Arabia, respectively, and 7.4% higher for Qatar (134). Such variations might be due to the differences in the literature search dates and languages, eligible sample size, GDM ascertainment criteria, and differences in the type of observational studies used for the prevalence estimation.

Our overall weighted GDM prevalence estimate depicted substantial heterogeneity (*I^2^*, 99.3%). This could be attributable to the less restrictive inclusion criteria in this review. In addition, the prevalence estimates of GDM can significantly differ with the variation in the GDM diagnostic criteria (135, 136). We noted clinical inconsistency in GDM diagnostic criteria used in the prevalence studies we reviewed (**Table 4**). This corresponds to the common use of existing nonuniform GDM diagnostic criteria in different countries (12, 134). Given the importance of the prevalence of GDM in meaningful intervention development, its estimation can be affected by the inclusion of studies that use different GDM diagnosing criteria (137, 138). The prevalence of GDM estimated based on the IADPSG criteria is usually high due to the low threshold for fasting blood glucose level relevant to other criteria. In our study, more than 25% of the studies used IADPSG criteria. To obtain homogenous and comparable prevalence estimates and to avoid confusion in practices of screening, diagnosis, and follow-up of GDM, health authorities should consider implementing uniform GDM diagnostic criteria nationally and across the MENA region.

The GDM prevalence estimates in our analysis suggested an increasing trend, parallel to the increase in BMI, correlating with the known fact that overweight and obesity are risk factors of GDM (139, 140). Although this does not prove a causal link between these parameters, it inevitably might significantly reflect the impact of the high burden of overweight and obesity in several countries in the MENA region, such as Egypt and the six GCC countries (141). This highlights the importance of investigating dietitians’ role in ensuring the appropriate caloric intake of GDM patients based on their BMI as per the recommendations of the ADA (142) and promoting exercise, especially among those with increased BMI (143).

GDM can have devastating maternal and birth consequences. Mothers with GDM are at higher risk of developing T2DM, dying, and undergoing C-section (23, 24, 144). Children born to mothers with untreated GDM face an increased risk of neonatal death and long-term disability (145, 146). Notably, diabetes in pregnancy is a neglected cause of maternal mortality globally, affecting one of every sixth pregnancy in the world, and some of the known GDM morbidities that may cause maternal death are postpartum hemorrhage, obstructed labor, and preeclampsia (147). In our analysis, although the prevalence of GDM was higher (16.35%) in countries with high MMR (>100/100,000 live births), it was also substantial in countries with lower MMR (≤100/100,000 live births). Although this does not prove temporality, it highlights the importance of researching complications of GDM (if any) leading to maternal deaths, to help healthcare providers in the MENA region establish protocols to prevent these anticipated adversities. GCC countries with the highest GDM prevalence, as presented in this study, are also burdened with high T2DM (148). There is no doubt that controlling GDM would have multiple benefits in avoiding unfavorable health consequences for both mothers and their babies.

### Strengths, Implications, and Limitations

The strengths of our review included its comprehensive characterization of the burden of GDM among pregnant women in several MENA countries. The review provides several weighted estimates in different population groups of the pregnant women at national, subregional, and regional levels that could be used, in addition to future work, to guide the planning, implementation, and evaluation of programs to prevent and control GDM. The overall and national-based pooled prevalence estimates might help policy makers of the respective MENA countries to contrast and quantify the local burden of GDM and introduce better policy initiatives regarding the flow of resources and funds for GDM care and management. Moreover, the finding of higher GDM prevalence corresponding to higher BMI categories might help in developing BMI-specific dietary and exercise guidelines. Furthermore, health authorities and organizations in the region are encouraged to review and consider standardizing the GDM diagnostic criteria at least at the national levels to improve the measurability and comparability of GDM rates and burden across the country and over time. Since we found a wide range of GDM diagnostic criteria used in the MENA region, health organizations across this region might consider moving toward the use of uniform GDM diagnostic criteria to produce better comparable statistical estimates in the future. For instance, in the UAE, different hospitals within the country use different GDM screening and ascertainment criteria (12). Having different GDM diagnostic criteria will preclude understanding the exact burden of the GDM.

Limitations included that our review did not provide any prevalence estimate for about 29% of the MENA region countries, as no prevalence data were available. This might have compromised the comprehensiveness of our prevalence estimates at the regional level. Since we believe that this study is the first to determine the prevalence of GDM in the MENA region, a comparison with previous similar estimates was not possible. This study offers scarce help regarding the prevalence of GDM with its associated comorbidities, such as gestational hypertension, preterm birth, and traumatic vaginal delivery (149), and separate review articles are warranted. The prevalence of GDM can also vary depending on several sociodemographic and maternal characteristics as well as within [urban or rural setting (150, 151)] and between countries and regions; however, our study does not provide such distinction on the prevalence data. In some of the reviewed studies, detailed information on the methodology and GDM measurement procedures was missing, and this limits the category-based generalizability of the measured pooled GDM prevalence. For instance, the 3.35-times increase in the prevalence of GDM in studies reported before 2009 compared with studies reported after 2009 should be cautiously interpreted, as there was an overlap in the time period in 14 studies that tested 29,983 women. The various thresholds for fasting blood glucose level to diagnose GDM, applied on the several criteria considered from the studies, might suggest a bias in the estimated GDM prevalence. Unless estimated by rigorous comparable survey and testing methodology in individual population-based studies, the burden of GDM at the country, subregional, or regional level should not be interpreted as the burden of the measured outcomes at the population level. Moroever, this review did not explore the associations between various maternal and neaonatal characterstics and GDM. Therefore, future systematic reviews and meta-analyses studies focusing on the burden of GDM according to different maternal and neonatal characteristics as well as on the strength of association between various maternal characteristics and GDM are warranted.

## Conclusions

Pregnant women in the MENA region are burdened with a relatively high GDM prevalence. Particularly, in the GCC and North African countries, the observed high burden of GDM may be mainly driven by the high prevalence of several risk factors for DM including overweight and obesity, parity, and late maternal age. To avoid maternal and newborn consequences, vigilant risk factor prevention programs and screening and management programs are necessary in the context of GDM. Moreover, unifying the GDM screening and diagnostic criteria, at least at the country level, is warranted to understand the precise burden of GDM. In countries that lack GDM burden data, high-quality research and surveillance programs are also warranted.

## Data Availability Statement

The data sets used and/or analyzed in the current study and the supplementary information files are available from the corresponding author on reasonable request.

## Author Contributions

Conceptualization, RHA. Methodology, RHA, NMA and MSP. Software, RHA. Validation, RHA. Formal analysis, RHA, NMA, and LAA. Resources, RHA. Writing—original draft preparation, SS and MSP. Writing—review and editing, NMA, MSP, SS, and LAA. Supervision, RHA. Project administration, RHA. Funding acquisition, RHA. All authors contributed to the article and approved the submitted version.

## Funding

This systematic review was funded by the Summer Undergraduate Research Experience (SURE) PLUS-Grant of the United Arab Emirates University, 2017 (research grant 31M348). The funding source had no role in the study design, collection, analysis, or interpretation of the data, nor in writing or the decision to submit this article for publication. The corresponding author had full access to all the data in the study and had final responsibility for the decision to submit for publication.

## Conflict of Interest

The authors declare that the research was conducted in the absence of any commercial or financial relationships that could be construed as a potential conflict of interest.

## Publisher’s Note

All claims expressed in this article are solely those of the authors and do not necessarily represent those of their affiliated organizations, or those of the publisher, the editors and the reviewers. Any product that may be evaluated in this article, or claim that may be made by its manufacturer, is not guaranteed or endorsed by the publisher.

## Supplementary Material

Supplementary File 1 PRISMA checklist.

Supplementary File 2 Overall weighted prevalence of GDM in pregnant women in North African countries (Morocco, Algeria, Tunisia, Libya, Egypt) by pregnancy trimester, body mass index, study period, ascertainment methodology, rate of cesarean section deliveries, and maternal mortality.

Supplementary File 3 Overall weighted prevalence of GDM in pregnant women in GCC countries and Yemen (the UAE, Qatar, Saudi Arabia, Kuwait, Yemen, Oman, Bahrain) by pregnancy trimester, body mass index, study period, ascertainment methodology, rate of cesarean section deliveries, and maternal mortality.

Supplementary File 4 Overall weighted prevalence of GDM in pregnant women in Iran/Iraq by pregnancy trimester, body mass index, study period, ascertainment methodology, rate of cesarean section deliveries, and maternal mortality.

Supplementary File 5 Overall weighted prevalence of GDM in pregnant women in the Levant region (Jordan, Syria, Palestine, Lebanon) by pregnancy trimester, body mass index, study period, ascertainment methodology, rate of cesarean section deliveries, and maternal mortality.

Supplementary File 6 Univariate and multivariable meta-regression analyses to identify sources of heterogeneity in studies reporting on the prevalence of GDM in pregnant women by different measured characteristics.

Supplementary Figure 7 Funnel plots **(A)** and Egger’s publication bias plot **(B)** examining small-study effects on the pooled GDM prevalence among pregnant women in the MENA region, 2000–2019.

Supplementary Figure 8 Risk of bias assessment of the 102 reviewed research reports on GDM.
