# Supplementary material for: Prevalence of Gestational Diabetes Mellitus in the Middle East and North Africa, 2000–2019: A Systematic Review, Meta-Analysis, and Meta-Regression
Source: Front Endocrinol (Lausanne). 2021 Aug 26;12:668447. doi: 10.3389/fendo.2021.668447 (PMC8427302; doi:10.3389/fendo.2021.668447)
Supplement: Supplementary File 1 — PRISMA checklist. [file Image_1.pdf]

frontiers

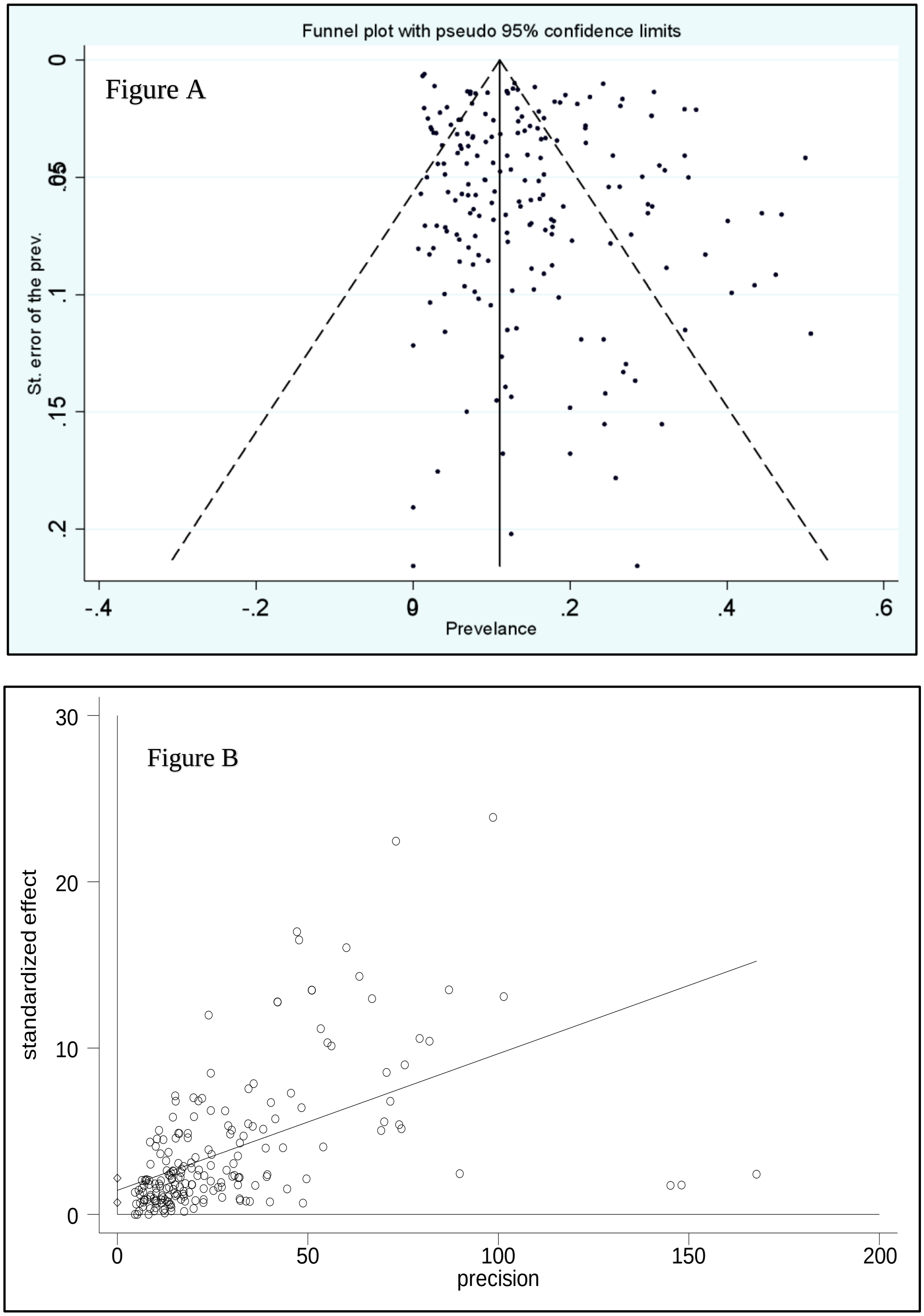

Supplementary Figure 1. Funnel plots (A) and Egger's publication bias plot (B) examining small-study effects on the pooled GDM prevalence among pregnant women in the MENA region, 2000–2019; Egger's test p<0·0001. Slope coefficient 0·082 (95% CI 0·06–0·10; p<0·001).

GDM, gestational diabetes mellitus.
