# Supplementary material for: Prevalence of Gestational Diabetes Mellitus in the Middle East and North Africa, 2000–2019: A Systematic Review, Meta-Analysis, and Meta-Regression
Source: Front Endocrinol (Lausanne). 2021 Aug 26;12:668447. doi: 10.3389/fendo.2021.668447 (PMC8427302; doi:10.3389/fendo.2021.668447)
Supplement: Supplementary File 2 — Overall weighted prevalence of GDM in pregnant women in North African countries (Morocco, Algeria, Tunisia, Libya, Egypt) by pregnancy trimester, body mass index, study period, ascertainment methodology, rate of cesarean section deliveries, and maternal mortality. [file Image_2.pdf]

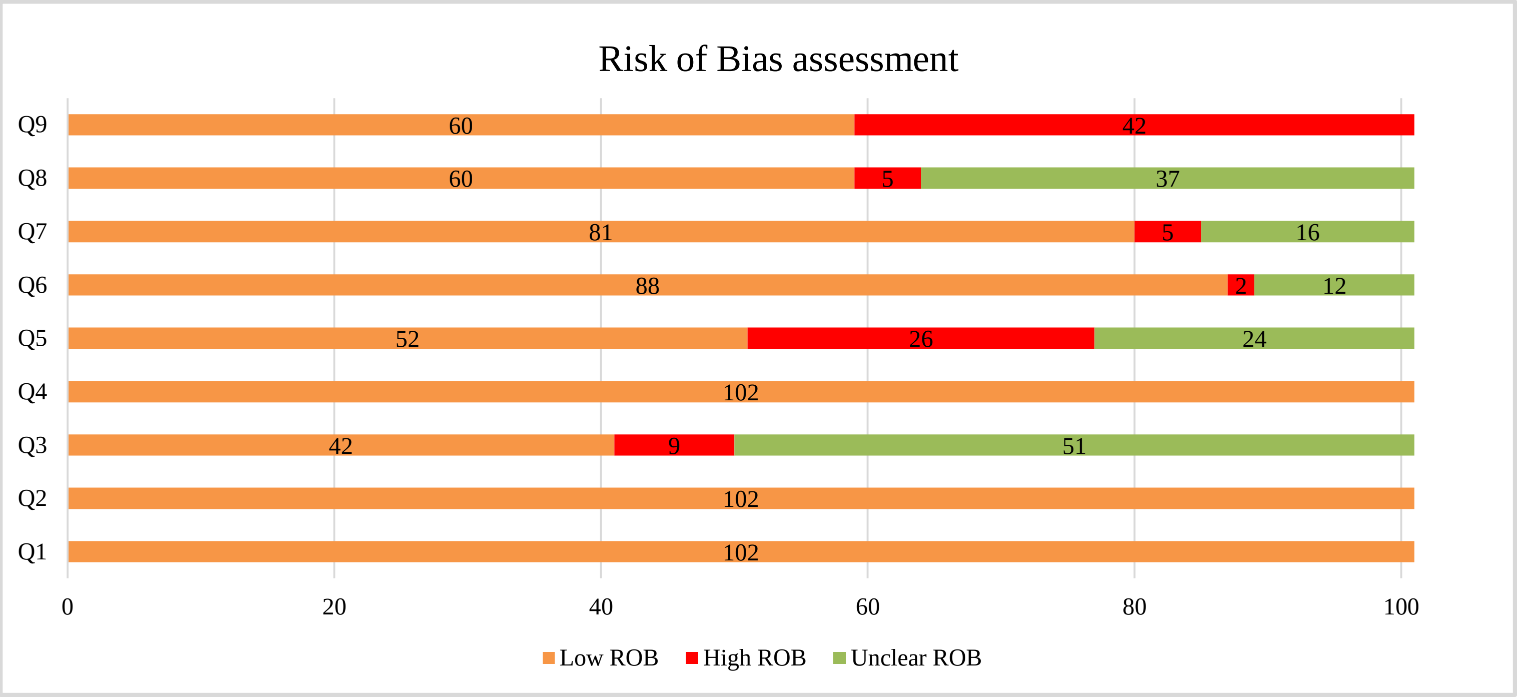

Supplementary Figure 2. Risk of Bias assessment of the 102 reviewed research reports on GDM. Average number of items clearly met: 6.7 out of the 9 Measure ROB items

Q1: Was the research question or objective in this paper clearly stated? 1: Low risk of bias (ROB), 2: High ROB, 3: Unclear ROB
Q2. Was the study population clearly specified and defined? 1: Low ROB, 2: High ROB, 3: Unclear ROB
Q 3. Was the participation rate of eligible persons at least 50%? 1: Low ROB, 2: High ROB, 3: Unclear ROB
Q4. Were all the subjects selected or recruited from the same or similar populations (including the same time period)? Were inclusion and exclusion criteria for being in the study prespecified and applied uniformly to all participants? 1: Low ROB, 2: High ROB, 3: Unclear ROB
Q5. Was a sample size justification, power description, or variance and effect estimates provided? 1: Low ROB, 2: High ROB, 3: Unclear ROB
Q6. Were the outcome measures (dependent variables) clearly defined, valid, reliable, and implemented consistently across all study participants? 1: Low ROB, 2: High ROB, 3: Unclear ROB

Q7: GDM ascertainment (1: biological assay/medical records; 2:self-reported; 3: unclear)
Q8: Sampling methodology (1: probability-based "random, consecutive, or whole population within a specified period of time"; 2: non–probability based; 3: unclear)
Q9: Precision (1: tested sample size ≥100; 2: tested sample size <100)
