# Supplementary material for: Prevalence of Gestational Diabetes Mellitus in the Middle East and North Africa, 2000–2019: A Systematic Review, Meta-Analysis, and Meta-Regression
Source: Front Endocrinol (Lausanne). 2021 Aug 26;12:668447. doi: 10.3389/fendo.2021.668447 (PMC8427302; doi:10.3389/fendo.2021.668447)
Supplement: Supplementary File 5 — Overall weighted prevalence of GDM in pregnant women in the Levant region (Jordan, Syria, Palestine, Lebanon) by pregnancy trimester, body mass index, study period, ascertainment methodology, rate of cesarean section deliveries, and maternal mortality. [file Table_3.docx]

**Supplementary Table 3.** Overall weighted prevalence of GDM in pregnant women in GCC countries and Yemen (UAE, Qatar, Saudi Arabia, Kuwait, Oman, Bahrain) by pregnancy trimester, body mass index, study period, ascertainment methodology, rate of caesarean section deliveries, and maternal mortality

|  | **No. of studies** | **Tested sample** | **GDM cases** | **GDM prevalence** | | | | **Heterogeneity measures** | | | |
| --- | --- | --- | --- | --- | --- | --- | --- | --- | --- | --- | --- |
|  |  |  |  | **Range (%)** | **Median (%)** | **Weighted prevalence (%)** | **95% CI** | **Q (*p* value)**^1^ | ***I*^2^ (%)^2^** | **95% prediction interval (%)^3^** | ***p* value^4^ (fixed)** |
| **Age** |  |  |  |  |  |  |  |  |  |  | <0.001 (<0.001) |
| 15–29 years | 15 | 16,328 | 2,711 | 0.0–29.9 | 13.7 | 11.8 | 8.1–16.1 | 722.3 (p<0.001) | 98.06 | 0.6–32.9 |  |
| ≥30 years | 18 | 20,904 | 5,399 | 4.4–50.7 | 26.6 | 23.4 | 19.4–27.6 | 723.2 (p<0.001) | 97.6 | 8.1–43.4 |  |
| Unclear age | 75 | 132,565 | 17,750 | 0.0–47.0 | 12.1 | 13.4 | 11.6–15.3 | 6,840.8 (p<0.001) | 98.9 | 1.9–32.6 |  |
| **Trimester** |  |  |  |  |  |  |  |  |  |  | 0.61 (<0.001) |
| First | 2 | 704 | 103 | 7.6–16.2 | 11.9 | 14.4 | 11.9–17.1 | – | – | – |  |
| Second | 47 | 86,668 | 11,676 | 0–36.1 | 12.1 | 13.6 | 11.6–15.6 | 3,041.6 (p<0.001) | 98.5 | 3.1–29.5 |  |
| Third | 7 | 9,948 | 577 | 2.7–50.7 | 20.2 | 20.2 | 9.0–34.7 | 613.1 (p<0.001) | 99.0 | 0.0–76.3 |  |
| Not reported | 52 | 72,477 | 13,504 | 0.0–47.0 | 15.5 | 15.3 | 12.7–18.1 | 4,772.5 (p<0.001) | 99.0 | 1.6–38.7 |  |
| **BMI** |  |  |  |  |  |  |  |  |  |  | <0.001 (<0.001) |
| Underweight | 1 | 67 | 0 | – | – | – | – | – | – | – |  |
| Normal weight | 8 | 2,891 | 273 | 1.0–29.2 | 5.4 | 7.3 | 2.8–13.5 | 189.3 (p<0.001) | 96.3 | 0.0–35.8 |  |
| Overweight | 6 | 2,121 | 299 | 4.3–35.1 | 9.9 | 12.5 | 5.0–22.5 | 178.1 (p<0.001) | 97.2 | 0.0–56.5 |  |
| Obese | 14 | 4,500 | 900 | 7.6–47 | 16.9 | 18.5 | 13.5–24.0 | 219.8 (p<0.001) | 94.5 | 2.9–42.9 |  |
| Unclear | 79 | 160,200 | 24,388 | 0.0–50.7 | 14.4 | 15.4 | 13.4–17.5 | 9,515.1 (p<0.001) | 99.2 | 2.3–36.8 |  |
| **Study period** |  |  |  |  |  |  |  |  |  |  | 0.19 (<0.001) |
| 2000–2009 | 29 | 48,227 | 6,161 | 0.0–50.7 | 12.0 | 11.9 | 8.5–15.8 | 3,683.6 (p<0.001) | 99.2 | 0.0–37.8 |  |
| 2010–2019 | 75 | 1121,292 | 19,667 | 2.1–47.0 | 14.7 | 15.9 | 14.0–17.9 | 6,136.2 (p<0.001) | 98.8 | 3.1–35.7 |  |
| Overlapping | 3 | 278 | 34 | 7.6–26.8 | 9.9 | 13.3 | 5.0–24.7 | 11.3 (p=0.004) | 82.2 | – |  |
| **Ascertainment** |  |  |  |  |  |  |  |  |  |  | <0.001 (<0.001) |
| WHO | 23 | 34,657 | 7,890 | 0.0–36.1 | 19.4 | 18.7 | 14.7–23.1 | 1,496.6 (p<0.001) | 98.9 | 3.0–43.3 |  |
| ADA | 12 | 23,2593 | 3,741 | 2.3–30.7 | 13.1 | 12.1 | 7.1–18.2 | 1,296.8 (p<0.001) | 99.3 | 0.0–40.3 |  |
| Self–reported | 2 | 1,345 | 44 | 2.9–4.4 | 3.7 | 3.2 | 2.3–4.2 | – | – | – |  |
| Medical records | 26 | 10,938 | 1,606 | 2.1–47.0 | 14.0 | 16.7 | 13.1–20.7 | 636.5 (p<0.001) | 96.1 | 2.3–40.0 |  |
| Unclear | 16 | 5,061 | 674 | 0.0–31.4 | 8.8 | 9.0 | 5.4–13.5 | 339.7 (p<0.001) | 95.6 | 0.0–32.5 |  |
| IADPSG | 13 | 29,518 | 4,961 | 2.7–50.7 | 18.7 | 21.9 | 14.7–30.0 | 2,881.8 (p<0.001) | 99.6 | 0.9–58.7 |  |
| Carpenter and Coustan | 2 | 2,701 | 415 | 10.2–21.9 | 16.1 | 14.9 | 13.6–16.3 | – | – | – |  |
| NDDG | 10 | 51,102 | 5,076 | 6.1–13.3 | 8.7 | 9.4 | 7.8–11.1 | 382.7 (p<0.001) | 97.6 | 4.0–16.7 |  |
| Fourth and Fifth International Workshop–Conference | 3 | 10,626 | 1,404 | 7.5–15.5 | 7.6 | 10.1 | 4.7–17.2 | – | – | – |  |
| NICE | 1 | 256 | 49 | – | – | 19.1 | 14.8–24.4 | – | – | – |  |
| **Rate of caesarean section** |  |  |  |  |  |  |  |  |  |  | <0.001 (<0.001) |
| <15% | 4 | 9,795 | 324 | 2.7–16.0 | 3.7 | 5.6 | 2.3–10.1 | 89.3 (p<0.001) | 96.6 | 0.0–36.5 |  |
| 15–29% | 102 | 157,498 | 25,493 | 0.0–50.7 | 14.2 | 15.5 | 13.8–17.1 | 7,661.9 (p<0.001) | 98.7 | 3.1–34.3 |  |
| >30% | 1 | 131 | 10 | – | – | 7.6 | 4.2–13.5 | – | – | – |  |
| Unclear | 1 | 2,373 | 33 | – | – | 1.4 | 1.0–1.9 | – | – | – |  |
| **Maternal mortality ratio** |  |  |  |  |  |  |  |  |  |  | <0.001 (<0.001) |
| ≤100 / 100,000 | 105 | 167,113 | 25,811 | 0.0–50.7 | 13.9 | 14.9 | 13.2–16.7 | 9,637.0 (p<0.001) | 98.9 | 2.2–35.6 |  |
| >100 / 100,000 | 1 | 311 | 16 | 5.1 | – | – | – | – | – | – |  |
| Unclear | 1 | 2,373 | 33 | – | – | 1.4 | 1.0–1.9 | – | – | – |  |
| **Overall**^5^ | 108 | 169,797 | 25,860 | 0.0–50.7 | 13.8 | 14.7 | 13.0–16.5 | 10,325.2 (p<0.001) | 99.0 | 1.9–36.0 |  |

Abbreviations: GDM, gestational diabetes mellitus; GCC, Gulf Cooperation Council countries. CI, confidence interval calculated using the exact binomial method; BMI, body mass index; WHO, World Health Organization; ADA, American Diabetes Association; IADPSG, International Association of Diabetes and Pregnancy Study Group; NDDG, National Diabetes Data Group; NICE, National Institute for Health and Care Excellence.

^1^Q: Cochran’s Q statistic is a measure assessing the existence of heterogeneity in estimates of GDM prevalence.

^2^ *I*^2^ is a measure assessing the percentage of between-study variation that is due to differences in GDM prevalence estimates across studies rather than chance.

^3^ Prediction intervals estimate the 95% confidence interval in which the true GDM prevalence estimate in a new study is expected to fall.

^4^ Estimating difference between subgroups using the random-effects model (fixed-effect model).

^5^ Excluding two studies in Yemen. Overall pooled estimate regardless of the tested population, sample size, and data collection period, using the most updated criteria when GDM ascertained using different criteria in the same population
