# Supplementary material for: Prevalence of Gestational Diabetes Mellitus in the Middle East and North Africa, 2000–2019: A Systematic Review, Meta-Analysis, and Meta-Regression
Source: Front Endocrinol (Lausanne). 2021 Aug 26;12:668447. doi: 10.3389/fendo.2021.668447 (PMC8427302; doi:10.3389/fendo.2021.668447)
Supplement: Supplementary File 6 — Univariate and multivariable meta-regression analyses to identify sources of heterogeneity in studies reporting on the prevalence of GDM in pregnant women by different measured characteristics. [file Table_4.docx]

**Supplementary Table 4.** Overall weighted prevalence of GDM in pregnant women in Iran and Iraq by pregnancy trimester, body mass index, study period, ascertainment methodology, rate of caesarean section deliveries, and maternal mortality

|  | **No. of studies** | **Tested sample** | **GDM cases** | **GDM prevalence** | | | | **Heterogeneity measures** | | | |
| --- | --- | --- | --- | --- | --- | --- | --- | --- | --- | --- | --- |
|  |  |  |  | **Range (%)** | **Median (%)** | **Weighted prevalence (%)** | **95% CI** | **Q (*p* value)**^1^ | ***I*^2^ (%)^2^** | **95% prediction interval (%)^3^** | ***p* value^4^ (fixed)** |
| **Age** |  |  |  |  |  |  |  |  |  |  | 0·07 (<0·001) |
| 15–29 years | 8 | 2,558 | 148 | 0·6–16·8 | 6·5 | 6·6 | 3·0–11·5 | 108·9 (*p*<0·001) | 93·6 | 0·0–28·8 |  |
| ≥30 years | 6 | 939 | 155 | 4·1–43·5 | 15·6 | 17·5 | 8·7–28·5 | 76·5 (*p*<0·001) | 93·5 | 0·0–61·9 |  |
| Unclear age | 53 | 28,842 | 2,903 | 0·0–50·0 | 8·3 | 11·4 | 8·9–14·0 | 2,267·3 (*p*<0·001) | 97·7 | 0·1–35·1 |  |
| **Trimester** |  |  |  |  |  |  |  |  |  |  | <0·001 (<0·001) |
| First | 8 | 4,926 | 270 | 2·2–37·2 | 7·0 | 8·3 | 4·6–13·0 | 191·6 (*p*<0·001) | 96·3 | 0·0–29·3 |  |
| Second | 27 | 16,387 | 1,751 | 0·0–50·0 | 8·8 | 12·0 | 8·6–15·9 | 1,274·6 (*p*<0·001) | 98·0 | 0·1–37·5 |  |
| Third | 10 | 4,094 | 761 | 6·9–43·5 | 19·4 | 20·2 | 13·5–27·9 | 265·5 (*p*<0·001) | 96·6 | 1·5–52·0 |  |
| Not reported | 22 | 6,932 | 424 | 0·6–28·6 | 6·9 | 7·4 | 5·0–10·1 | 274·5 (*p*<0·001) | 92·3 | 0·1–22·8 |  |
| **BMI** |  |  |  |  |  |  |  |  |  |  | 0·02 (<0·001) |
| Underweight | 1 | 27 | 0 | — | — | — | — | — | — | — |  |
| Normal weight | 3 | 931 | 62 | 5·9–20·0 | 6·0 | 8·3 | 3·6–14·7 | 8·6 (*p*=0·014) | 76·7 | — |  |
| Overweight | 1 | 381 | 35 | — | — | 9·2 | 6·7–12·5 | — | — | — |  |
| Obese | 2 | 182 | 27 | 11·3–16·7 | 14·0 | 14·7 | 9·8–20·3 | — | — | — |  |
| Unclear | 60 | 30,818 | 3,082 | 0·6–50·0 | 8·3 | 11·5 | 9·1–14·0 | 2,522·2 (*p*<0·001) | 97·7 | 0·1–35·6 |  |
| **Study period** |  |  |  |  |  |  |  |  |  |  | 0·08 (<0·001) |
| 2000–2009 | 16 | 7,343 | 492 | 2·2–24·4 | 7·4 | 8·2 | 5·9–11·0 | 215·3 (*p*<0·001) | 93·0 | 0·8–21·9 |  |
| 2010–2019 | 42 | 23,608 | 2,548 | 0·0–50·0 | 8·8 | 11·9 | 8·9–15·2 | 2,180·2 (*p*<0·001) | 98·1 | 0·0–38·7 |  |
| Overlapping | 9 | 1,388 | 166 | 5·9–28·6 | 13·5 | 13·5 | 8·2–19·7 | 67·8 (*p*<0·001) | 88·2 | 0·3–38·4 |  |
| **Ascertainment**^9^ |  |  |  |  |  |  |  |  |  |  | <0·001 (<0·001) |
| ADA | 12 | 7,554 | 1,057 | 6·9–37·2 | 14·1 | 15·4 | 11·1–20·3 | 304·7 (*p*<0·001) | 96·4 | 2·2–37·2 |  |
| ADA/IADPSG | 1 | 574 | 287 | — | — | 50·0 | 45·9–54·1 | — | — | — |  |
| Self–reported | 1 | 62 | 7 | — | — | 11·3 | 5·6–21·5 | — | — | — |  |
| Medical records | 14 | 9,710 | 382 | 0·6–25·8 | 4·0 | 4·7 | 3·2–6·5 | 142·8 (*p*<0·001) | 90·9 | 0·3–12·9 |  |
| Unclear | 16 | 4,212 | 349 | 2·6–28·6 | 8·0 | 9·1 | 6·5–12·1 | 120·2 (*p*<0·001) | 87·5 | 1·1–22·8 |  |
| IADPSG | 5 | 2,479 | 493 | 10·0–43·5 | 22·0 | 23·6 | 13·9–34·9 | 139·5 (*p*<0·001) | 97·1 | 0·0–70·2 |  |
| Carpenter and Coustan | 11 | 5,767 | 340 | 2·2–24·4 | 7·0 | 7·4 | 5·1–9·9 | 102·2 (*p*<0·001) | 90·2 | 1·0–18·2 |  |
| Fourth and Fifth International Workshop–Conference | 3 | 702 | 191 | 7·3–44·4 | 29·9 | 25·3 | 6·8–50·5 | 100·2 (*p*<0·001) | 98·0 | — |  |
| ACOG | 4 | 1,279 | 100 | 0·0–16·7 | 7·6 | 7·7 | 3·7–12·9 | 18·11 (*p*<0·001) | 83·4 | 0·0–36·8 |  |
| **Rate of caesarean section** |  |  |  |  |  |  |  |  |  |  | 0·30 (<0·001) |
| 15–29% | 5 | 2,720 | 345 | 2·5–24·5 | 13·3 | 11·5 | 5·7–18·8 | 33·3 (*p*<0·001) | 88·0 | 0·0–43·2 |  |
| >30% | 59 | 27,203 | 2,747 | 0·0–50·0 | 8·3 | 11·4 | 9·0–14·1 | 2,345·8 (*p*<0·001) | 97·5 | 0·0–36·3 |  |
| Unclear | 3 | 2,416 | 114 | 2·2–15·0 | 5·6 | 6·5 | 2·1–12·9 | 50·2 (*p*<0·001) | 96·0 | — |  |
| **Maternal mortality** |  |  |  |  |  |  |  |  |  |  | 0·12 (<0·001) |
| ≤100/100,000 | 64 | 29,923 | 3,092 | 0·0–50·0 | 8·8 | 11·5 | 9·2–13·9 | 2,418·9 (*p*<0·001) | 97·4 | 0·1–35·3 |  |
| Unclear | 3 | 2,416 | 114 | 2·2–15·0 | 5·6 | 6·5 | 2·1–12·9 | 50·2 (*p*<0·001) | 96·0 | — |  |
| **Overall^5^** | 67 | 32,339 | 3,206 | 0·0–50·0 | 8·3 | 11·2 | 9·0–13·5 | 2,549·2 (*p*<0·001) | 97·4 | 0·1–34·5 |  |

Abbreviations: CI, confidence interval calculated using the exact binomial method; GDM, gestational diabetes mellitus; BMI, body mass index; ADA, American Diabetes Association; IADPSG, International Association of Diabetes and Pregnancy Study Group; ACOG, American College of Obstetricians and Gynecologists.

^1^ Q: Cochran’s Q statistic is a measure assessing the existence of heterogeneity in estimates of GDM prevalence.

^2^ *I*^2^ is a measure assessing the percentage of between-study variation that is due to differences in GDM prevalence estimates across studies rather than chance.

^3^ Prediction intervals estimate the 95% confidence interval in which the true GDM prevalence estimate in a new study is expected to fall.

^4^ Estimating difference between sub-groups using the random-effects model (fixed effect model)

^5^ Overall pooled estimate regardless of the tested population, sample size, and data collection period, using the most updated criteria when GDM is ascertained using different criteria in the same population.
