# Supplementary material for: Prevalence of Gestational Diabetes Mellitus in the Middle East and North Africa, 2000–2019: A Systematic Review, Meta-Analysis, and Meta-Regression
Source: Front Endocrinol (Lausanne). 2021 Aug 26;12:668447. doi: 10.3389/fendo.2021.668447 (PMC8427302; doi:10.3389/fendo.2021.668447)
Supplement: Supplementary Figure 7 — Funnel plots (A) and Egger’s publication bias plot (B) examining small-study effects on the pooled GDM prevalence among pregnant women in the MENA region, 2000–2019. [file Table_5.docx]

**Supplementary table 5.** Overall weighted prevalence of GDM in pregnant women in the Levant region (Jordan, Lebanon) by pregnancy trimester, body mass index, study period, ascertainment methodology, rate of caesarean section deliveries, and maternal mortality

|  | **No. of studies** | **Tested sample** | **GDM cases** | **GDM prevalence** | | | | **Heterogeneity measures** | | | |
| --- | --- | --- | --- | --- | --- | --- | --- | --- | --- | --- | --- |
|  |  |  |  | **Range (%)** | **Median (%)** | **Weighted prevalence (%)** | **95% CI** | **Q (*p* value)**^1^ | ***I*^2^ (%)^2^** | **95% prediction interval (%)^3^** | ***p* value^4^ (fixed)** |
| **Age** |  |  |  |  |  |  |  |  |  |  | <0·001 (<0·001) |
| 15–29 years | 1 | 301 | 24 | — | — | 8·0 | 5·4–11·6 | — | — | — |  |
| ≥30 years | 2 | 343 | 63 | 16·6–31·7 | 24·1 | 17·9 | 13·9–22·2 | — | — | — |  |
| Unclear age | 5 | 43,414 | 540 | 1·2–15·4 | 1·5 | 1·8 | 1·1–2·6 | 47·8 (*p*<0·001) | 91·6 | 0·2–4·8 |  |
| **Trimester** |  |  |  |  |  |  |  |  |  |  | <0·001 (<0·001) |
| Second | 3 | 644 | 87 | 1·5–31·7 | 12·1 | 16·3 | 7·4–27·8 | 20·0 (*p*<0·001) | 90·0 | — |  |
| Third | 1 | 104 | 16 | – | – | 15·4 | 9·7–23·5 | — | — | — |  |
| Not reported | 4 | 43,310 | 524 | 1·2–34·0 | 6·7 | 1·1 | 0·8–1·4 | 11·0 (*p*=0·012) | 72·6 | 0·2–2·5 |  |
| **BMI** |  |  |  |  |  |  |  |  |  |  | – |
| Unclear | 8 | 44,058 | 627 | 1·2–31·7 | 7·3 | 5·8 | 3·9 –7·9 | 239·7 (*p*<0·001) | 97·1 | 1·0–13·7 |  |
| **Study period** |  |  |  |  |  |  |  |  |  |  | – |
| 2010–2019 | 8 | 44,058 | 627 | 1·2–31·7 | 7·3 | 5·8 | 3·9 –7·9 | 239·7 (*p*<0·001) | 97·1 | 1·0–13·7 |  |
| **Ascertainment**^9^ |  |  |  |  |  |  |  |  |  |  | <0·001 (<0·001) |
| Self–reported | 1 | 107 | 7 | — | — | 6·5 | 3·2–12·9 | — | — | — |  |
| Medical records | 1 | 21,075 | 253 | — | — | 1·2 | 1·1–1·4 | — | — | — |  |
| Unclear | 2 | 22,128 | 264 | 1·2–1·5 | 1·3 | 1·1 | 0·9–1·2 | — | — | — |  |
| IADPSG | 4 | 748 | 103 | 8·0–31·7 | 16·0 | 15·8 | 8·9–24·2 | 20·5 (*p*<0·001) | 85·4 | 0·0–59·4 |  |
| **Rate of caesarean section** |  |  |  |  |  |  |  |  |  |  | 0·01 (<0·001) |
| 15–29% | 6 | 43,847 | 604 | 1·2–31·7 | 4·7 | 4·7 | 3·0–6·7 | 193·7 (*p*<0·001) | 97·4 | 0·4–12·5 |  |
| >30% | 2 | 211 | 23 | 6·5–15·4 | 11·0 | 10·5 | 6·7–15·1 | — | — | — |  |
| **Maternal mortality** |  |  |  |  |  |  |  |  |  |  | — |
| ≤100/100,000 | 8 | 44,058 | 627 | 1·2–31·7 | 7·3 | 5·8 | 3·9–7·9 | 239·7 (*p*<0·001) | 97·1 | 1·0–13·7 |  |
| **Overall^5^** | 8 | 44,058 | 627 | 1·2–31·7 | 7·3 | 5·8 | 3·9–7·9 | 239·7 (*p*<0·001) | 97·1 | 1·0–13·7 |  |

Abbreviations: CI, confidence interval calculated using the exact binomial method; GDM, gestational diabetes mellitus; ; BMI, body mass index; IADPSG, International Association of Diabetes and Pregnancy Study Group.

^1^ Q: Cochran’s Q statistic is a measure assessing the existence of heterogeneity in estimates of GDM prevalence.

^2^ *I*^2^ is a measure assessing the percentage of between-study variation that is due to differences in GDM prevalence estimates across studies rather than chance.

^3^ Prediction intervals estimate the 95% confidence interval in which the true GDM prevalence estimate in a new study is expected to fall.

^4^ Estimating difference between subgroups using the random-effects model (fixed-effect model).

^5^ Overall pooled estimate regardless of the tested population, sample size, and data collection period, using the most updated criteria when GDM is ascertained using different criteria in the same population.
