# Supplementary material for: Prevalence of Gestational Diabetes Mellitus in the Middle East and North Africa, 2000–2019: A Systematic Review, Meta-Analysis, and Meta-Regression
Source: Front Endocrinol (Lausanne). 2021 Aug 26;12:668447. doi: 10.3389/fendo.2021.668447 (PMC8427302; doi:10.3389/fendo.2021.668447)
Supplement: Supplementary Figure 8 — Risk of bias assessment of the 102 reviewed research reports on GDM. [file Table_6.docx]

**Supplementary Table 6.** Univariate and multivariable meta-regression analyses to identify sources of heterogeneity in studies reporting on GDM prevalence in pregnant women by different measured characteristics

|  | **Univariate analyses** | | **Multivariable analyses** | |
| --- | --- | --- | --- | --- |
|  | **OR (95% CI)** | ***p* value*** | **aOR (95% CI)** | ***p* value^1^** |
| **Country** |  |  |  |  |
| Saudi Arabia | 1·00 | – | 1·00 | – |
| Algeria | 0·25 (0·04-1·62) | 0·146 | 0·28 (0·05-1·56) | 0·148 |
| Bahrain | 0·89 (0·46-1·70) | 0·722 | 0·70 (0·38-1·31) | 0·401 |
| Egypt | 0·95 (0·42-2·16) | 0·906 | 0·68 (0·26-1·75) | 0·422 |
| Lebanon | 0·92 (0·24-3·51) | 0·904 | 0·96 (0·27-3·37) | 0·952 |
| Libya | 0·12 (0·02-0·77) | 0·025 | 0·09 (0·02-0·52) | 0·007 |
| Morocco | 2·34 (1·36-4·03) | 0·002 | 2·22 (1·30-3·76) | 0·003 |
| Iran | 0·91 (0·61-1·37) | 0·667 | 0·76 (0·51-1·13) | 0·175 |
| Iraq | 0·97 (0·61-1·38) | 0·943 | 0·65 (0·23-1·80) | 0·401 |
| Jordan | 0·42 (0·18-0·95) | 0·037 | 0·38 (0·18-0·80) | 0·011 |
| Oman | 0·82 (0·45-1·49) | 0·514 | 0·70 (0·38-1·27) | 0·236 |
| Qatar | 1·88 (1·1-3·23) | 0·022 | 2·35 (1·39-3·95) | 0·001 |
| United Arab Emirates | 1·18 (0·65-2·15) | 0·572 | 1·26 (0·72-2·22) | 0·413 |
| Sudan | 2·24 (0·74-6·82) | 0·151 | 2·00 (0·73-5·54) | 0·177 |
| Tunisia | 0·83 (0·13-5·35) | 0·847 | 0·94 (0·17-5·16) | 0·951 |
| **Age** |  |  |  |  |
| 15–29 years | 1·00 | — | 1·00 |  |
| ≥30 years | 2·82 (1·64-4·83) | <0·001 | 2·52 (1·51-4·21) | <0·001 |
| Unclear age | 1·34 (0·88-2·04) |  | 2·02 (1·30-3·17) | 0·002 |
| **Trimester** |  |  |  |  |
| First | 1·00 | — | 1·00 | – |
| Second | 1·60 (0·87-2·97) | 0·133 | 1·46 (0·82-2·60) | 0·199 |
| Third | 2·64 (1·26-5·51) | 0·010 | 1·20 (0·50-2·87) | 0·675 |
| Not reported | 1·39 (0·75-2·59) | 0·288 | 1·01 (0·56-1·82) | 0·965 |
| **BMI** |  |  |  |  |
| Normal weight | 1·00 | — | 1·00 | — |
| Overweight | 1·72 (0·68-4·40) | 0·250 | 1·71 (0·76-3·82) | 0·192 |
| Obese | 2·73 (1·28-5·83) | 0·009 | 2·92 (1·50-5·69) | 0·002 |
| Unclear | 1·90 (1·04-3·49) | 0·036 | 2·34 (1·32-4·17) | 0·004 |
| **Sample size** |  |  |  |  |
| <100 | 1·00 | — | 1·00 | — |
| ≥100 | 0·65 (0·43-0·97) | 0·037 | 0·58 (0·01-0·11) | 0·003 |

Abbreviations: GDM, gestational diabetes mellitus; OR, odds ratio; aOR, adjusted odds ratio; CI, confidence interval calculated using the “exact” method; BMI, body mass index.

GDM: gestational diabetes mellitus * Statistically significant at *p*<0·1.

^1^ Statistically significant at *p*<0·05.
